# Ellagic acid improves benign prostate hyperplasia by regulating androgen signaling and STAT3

**DOI:** 10.1038/s41419-022-04995-3

**Published:** 2022-06-17

**Authors:** Woo Yong Park, Gahee Song, Ja Yeon Park, Kwang Seok Ahn, Hyun Jeong Kwak, Jinbong Park, Jun Hee Lee, Jae-Young Um

**Affiliations:** 1 Department of Science in Korean Medicine, Graduate School, Kyung Hee University, Seoul, Republic of Korea; 2 Department of Pharmacology, College of Korean Medicine, Kyung Hee University, Seoul, Republic of Korea; 3 Department of Life Science, College of Natural Sciences, Kyonggi University, Suwon, Republic of Korea; 4 Department of Sasang Constitutional Medicine, College of Korean Medicine, Kyung Hee University, Seoul, Republic of Korea

**Keywords:** Prostatic diseases, Pharmacology, Experimental models of disease

## Abstract

Benign prostate hyperplasia (BPH) is an age-related disease in men characterized by the growth of prostate cells and hyperproliferation of prostate tissue. This condition is closely related to chronic inflammation. In this study, we highlight the therapeutic efficacy of ellagic acid (EA) for BPH by focusing on the AR signaling axis and STAT3. To investigate the effect of EA on BPH, we used EA, a phytochemical abundant in fruits and vegetables, to treat testosterone propionate (TP)-induced BPH rats and RWPE-1 human prostate epithelial cells. The EA treatment reduced prostate weight, prostate epithelial thickness, and serum DHT levels in the TP-induced BPH rat model. In addition, EA improved testicular injury by increasing antioxidant enzymes in testis of the BPH rats. EA reduced the protein levels of AR, 5AR2, and PSA. It also induced apoptosis by regulating Bax, Bcl_xL, cytochrome c, caspase 9, and caspase 3 with increasing mitochondrial dynamics. Furthermore, EA reduced the expression of IL-6, TNF-α, and NF-κB, as well as phosphorylation of STAT3 and IκBα. These findings were also confirmed in TP-treated RWPE-1 cells. Overall, our data provide evidence of the role of EA in improving BPH through inhibition of AR and the STAT3 pathway.

## Introduction

Ellagic acid (EA) is a natural phenolic compound and previous studies have previously suggested the potential of EA as an alternative therapeutic agent for diseases such as obesity, diabetes, atherosclerosis, and cancer [1, 2]. Its beneficial effects are induced through the regulation of multiple pathways such as anti-inflammation, anti-oxidative, and apoptosis [1]. However, the therapeutic potential of EA against BPH has not been supported.

Benign prostatic hyperplasia (BPH) is the condition of an enlarged prostate that is caused by accelerated proliferation of stromal and epithelial cells, but which does not induce tumor production [3]. Abnormal overgrowth of the prostate causes various histological changes, such as increased epithelial thickness and penetration of epithelial tissue into the lumen region, leading to urinary diseases such as lower urinary tract symptoms (LUTS), acute urinary retention (AUR), and bladder outlet obstruction (BOO) [4]. Most BPH patients are >50 years of age and are known to have lower quality of life due to BPH-induced symptoms such as urinary intermittency, incomplete emptying, weak stream, staining, urgency, and nocturia [5].

The pathological cause of BPH is an imbalance between testosterone and dihydrotestosterone (DHT), which is a critical factor in the pathologic diagnosis of BPH [6]. Testosterone, a major androgen that plays an important role in development of the male reproductive system [7], is converted to DHT by action of type 1 or type 2 5α-reductase. The DHT activates androgen receptor (AR)-mediated prostate cell proliferation and consequentially causes prostate overgrowth [8]. In addition to hormonal imbalance, inflammation is also the pathological cause of BPH [6]. The prostate of a BPH patient shows extensive inflammation, especially pro-inflammatory mediators such as interleukin (IL)−6 and tumor necrosis factor (TNF)-α. Moreover, nuclear factor kappa-light-chain-enhancer from activated B cells (NF-kB) increased in the prostate tissue of BPH patients [9, 10].

The pharmacological treatment of BPH has been focused on reducing symptoms and prostatic growth using agents of α−1 adrenergic receptor antagonists (α1-blockers) and 5α-reductase inhibitors (5ARIs) [11]. Finasteride is one of the most prescribed drugs to treat BPH and blocks testosterone from converting to DHT by inhibiting type 2 5α-reductase activity [12]. However, long-term treatment with finasteride has been reported to have numerous adverse effects, including erectile dysfunction, depression, or loss of libido [13]. Furthermore, accumulated evidence suggests that non-steroidal anti-inflammatory drugs (NSAIDs) might have a beneficial effect on the development and progression of BPH in experimental BPH models, but proof of the efficacy of NSAIDs in the treatment of BPH requires further clinical study [14].

Thus, the aim of the present study was to explore the effect and the underlying mechanisms of EA on BPH development using testosterone propionate (TP)-induced BPH rats and RWPE-1 human prostate epithelial cell lines.

## Results

### EA reduces androgen-mediated prostate hyperplasia in a TP-induced BPH rat model

**Fig. 1:**
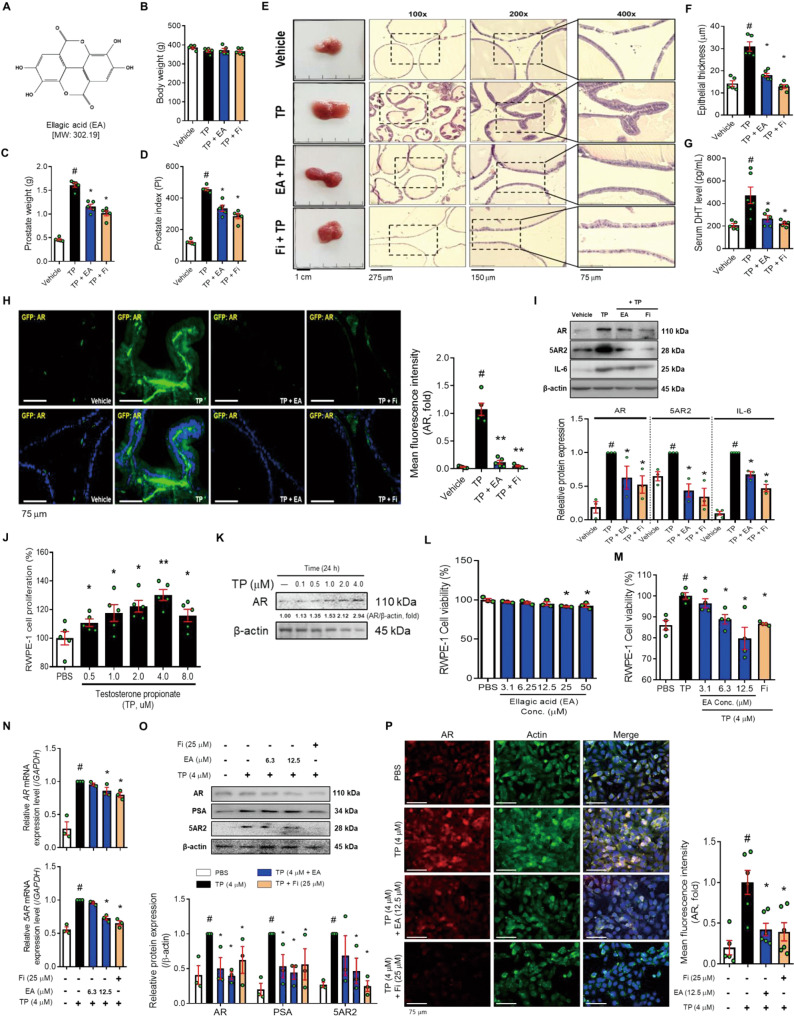
EA suppresses prostatic hyperplasia by inhibition of 5AR-AR signaling axis in TP-induced BPH SD rats and TP-treated RWPE-1 cells. **A** Chemical structure of EA, **B** Body, and **C** Prostate weights of vehicle, TP (5 mg/kg**/**day), TP + EA (20 mg/kg**/**day), and TP + Fi (1 mg/kg**/**day)-injected rats were measured (*n* = 6 per group), **D** Prostate index calculated by prostate weight (mg)/body weight (100 g), **E** Representative images of a prostate and paraffin-embedded prostate stained with H&E (magnification 100×, scale bar 275 μm, 200×, scale bar 150 μm, and 400×, scale bar 75 μm), **F** Prostate epithelial thickness measured using ImageJ software, **G** Serum level of DHT measured using a DHT ELISA kit, **H** AR (green) and nuclei (blue) were detected in prostate tissue of rats by immunofluorescence staining (400× magnification, scale bar 75 μm). **I** Protein levels of AR, 5AR2, and IL-6 were analyzed by Western blot. The loading control was β-actin. **J** Cell cytotoxicity of TP was measured by WST-1 assay. **K** Protein level of AR was measured by Western blot analysis. **L**, **M** Cell viability of EA on TP (4.0 μM)-treated or non-treated RWPE-1 cells was measured by WST-1 assay, and Fi (25 μM) was used as a positive control. **N** mRNA expressions of *AR* and *5AR* were measured by RT-PCR and data were normalized to *GAPDH*. **O** Protein levels of AR, 5AR2, and PSA were analyzed by Western blot analysis. **P** AR (red), Actin (green) and nuclei (blue) were detected by immunofluorescence staining (400× magnification, scale bar 75 μm). β-actin was used as a loading control. All data are expressed as the mean ± S.E.M. of data from three or more separate experiments. Statistical differences were evaluated using the unpaired *t*-test and a subsequent post-hoc one-tailed Mann–Whitney *U*-test. ^#^*p* < 0.05 vs. vehicle group or PBS-treated RWPE-1 cells, ^*^p < 0.05 vs. TP group or TP-treated RWPE-1 cells. PBS phosphate-buffered saline, TP testosterone propionate, EA ellagic acid, Fi finasteride.

The chemical structure of EA is shown in Fig. 1A. As shown in Fig. 1B, the difference in body weight between TP injection groups was not significant. The prostate in the TP group increased 3.5-fold over that in the vehicle group. In contrast, groups of rats treated with EA or Fi showed a reduction of prostate weight (Fig. 1C). Prostate index (PI) used to express the mass of the prostate relative to body weight was calculated using the prostate mass (mg)/body weight (100 g). The PI showed similar results to prostate tissue weight (Fig. 1D). To observe the histological changes of the prostate, prostates were stained with hematoxylin and eosin dye (Fig. 1E). The thickness of the prostate epithelial tissue in the EA or Fi treatment group was significantly reduced compared to the TP treatment group (Fig. 1F).

The serum DHT from TP-injected rats was increased 2.4-fold over the vehicle group. However, the serum DHT in groups of rats treated with EA or Fi decreased respectively (Fig. 1G). We then examined the protein levels of AR, 5AR2, and IL-6 in the prostate. The expression of AR was detected in the prostates by Immunofluorescence staining. As a result, the groups of rats treated with EA or Fi showed decreased AR expression in the prostate compared to the groups of rats treated with TP (Fig. 1H). The protein levels of AR, 5AR2, and IL-6 in the prostate were significantly reduced in the groups of rats treated with EA or Fi, compared to the groups of rats treated with TP (Fig. 1I). In addition, to investigate the effect of EA on liver and kidney injuries, we confirmed the levels of ALT, AST, creatinine, and BUN in the serum of the rats, and the results were expressed in Supplementary Fig. 1A, B.

### EA regulates androgen-mediated proliferation through AR signaling axis in TP-treated RWPE-1 cells

To establish an in vitro experimental model of BPH, we used RWPE-1 cells. We treated with various concentrations of TP (0.5–8.0 μM) to investigate how TP treatment affects cell proliferation by RWPE-1 cells. The cell proliferation increased after 4.0 μM of TP treatment (Fig. 1J). We further investigated whether treatment with 1.0–4.0 μM of TP affects protein levels of AR in RWPE-1 cells. The TP (4.0 μM) increased AR levels compared to PBS-treated cells (Fig. 1K). Therefore, the following experiments were conducted at a TP concentration of 4.0 μM.

Because EA showed no cytotoxicity at concentrations of <12.5 μM (Fig. 1L), we investigated whether EA (3.1–12.5 μM) could inhibit proliferation in RWPE-1 cells. Fi (25 μM) was used as a positive control. An increased cell proliferation of RWPE-1 cells by TP was significantly reduced by the treatment of EA or Fi (Fig. 1M). EA (12.5 μM) or Fi treatment substantially reduced the mRNA expression of AR and 5AR in the AR signaling pathway (Fig. 1N). Furthermore, EA or Fi treatment reduced the protein levels of androgen-mediated growth factors (AR, 5AR, and PSA) in TP-treated RWPE-1 cells (Fig. 1O). Cytological expression of AR (green dye) was decreased by EA or Fi treatment of TP-treated RWPE-1 cells (Fig. 1P).

### EA restores testicular injury by increasing catalase activity and decreasing MDA level in a TP-induced BPH rat model

**Fig. 2:**
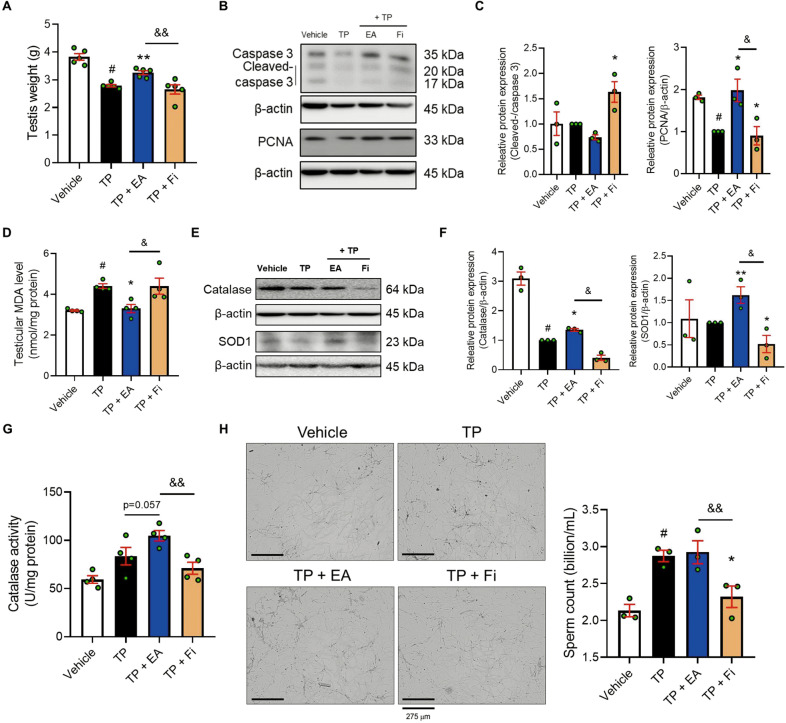
EA alleviates testicular injury and epididymal sperm loss in TP-induced BPH SD rats. **A** Testis weights of vehicle, TP (5 mg/kg**/**day), TP + EA (20 mg/kg**/**day), and TP + Fi (1 mg/kg**/**day)-injected rats were measured (*n* = 5 per group). **B**, **C** Protein levels of pro/cleaved-caspase 3 and PCNA were analyzed by Western blot analysis. **D** MDA level in testis was measured using ELISA kit. **E**, **F** Protein levels of catalase and SOD1 were analyzed by Western blot analysis. **G** Catalase activity in testis was measured using ELISA kit. **H** Representative images of epididymal sperm (magnification 100×, scale bar 275 μm) were expressed, and sperm count was measured using ImageJ software. The loading control was β-actin. All data are expressed as the mean ± S.E.M. of data from three or more separate experiments. Statistical differences were evaluated using the unpaired *t*-test and a subsequent post-hoc one-tailed Mann–Whitney *U*-test. ^#^*p* < 0.05 vs. vehicle group, ^*^p < 0.05 and ^**^p < 0.01 vs. TP group, ^&^*p* < 0.05 and ^&&^*p* < 0.01 vs. Fi group. TP testosterone propionate, EA ellagic acid, Fi finasteride.

Interestingly, we found that the testis weight was reduced in the TP-induced BPH rats, and in contrast to the Fi group, the testis weight was restored in the EA group (Fig. 2A). To investigate the protective effect of EA on testis weight loss in the TP-induced BPH rats, we confirmed apoptosis and proliferation markers in the testis. As shown in Fig. 2B, C, the decreased PCNA protein level in the testis of BPH rats was restored to normal level by EA treatment. In contrast, cleaved-caspase 3/caspase 3 ratio were increased by Fi treatment in the testis, but EA did not affect the marker. Because oxidative stress is one of the major factors in tissue injury [15], we also verified the lipid peroxidation process-related factors: antioxidant enzymes (catalase and SOD1) and an end product of the lipid peroxidation process (malondialdehyde, MDA), in the testis. The MDA level of the testis in the TP group was significantly increased compared to the vehicle group. However, the increased MDA level in the testis was reduced by EA treatment (Fig. 2D). In contrast to the Fi group, the protein levels of catalase and SOD1 in the testis of the EA group were higher than for the TP group (Fig. 2E–G). Furthermore, to investigate the effect of EA on sperm, we measured the number of sperm in the epididymis of each group. No difference was observed between the TP and EA groups. However, Fi treatment decreased the sperm count (Fig. 2H).

### EA increases mitochondrial dynamics and induces apoptosis in a TP-induced BPH rat model and TP-treated RWPE-1 cells

**Fig. 3:**
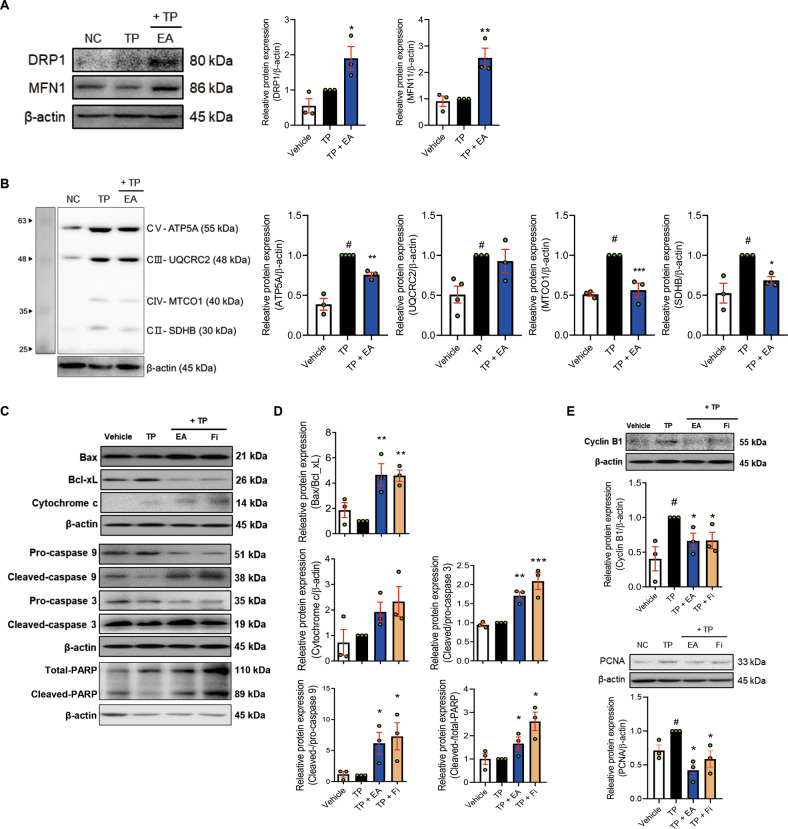
EA induces mitochondrial dynamics regulation and apoptosis in prostate of TP-induced BPH SD rats. **A**, **B** Protein levels of DRP1, MFN1, and OXPHOS were measured by Western blot analysis. **C**, **D** Protein levels of Bax, Bcl_xL, cytochrome c, pro/cleaved-caspase 9, pro/cleaved-caspase 3, and total/cleaved-PARP were analyzed by Western blot analysis. **E** Protein levels of cyclin B1 and PCNA was measured by Western blot analysis. β-actin was used as a loading control. All data are expressed as the mean ± S.E.M. of data from three or more separate experiments. Statistical differences were calculated by one-way ANOVA with post-hoc Tukey’s test. ^#^*p* < 0.05 vs. vehicle group, ^*^p < 0.05 and ^**^p < 0.01 vs. TP group. TP testosterone propionate, EA ellagic acid, Fi finasteride.

**Fig. 4:**
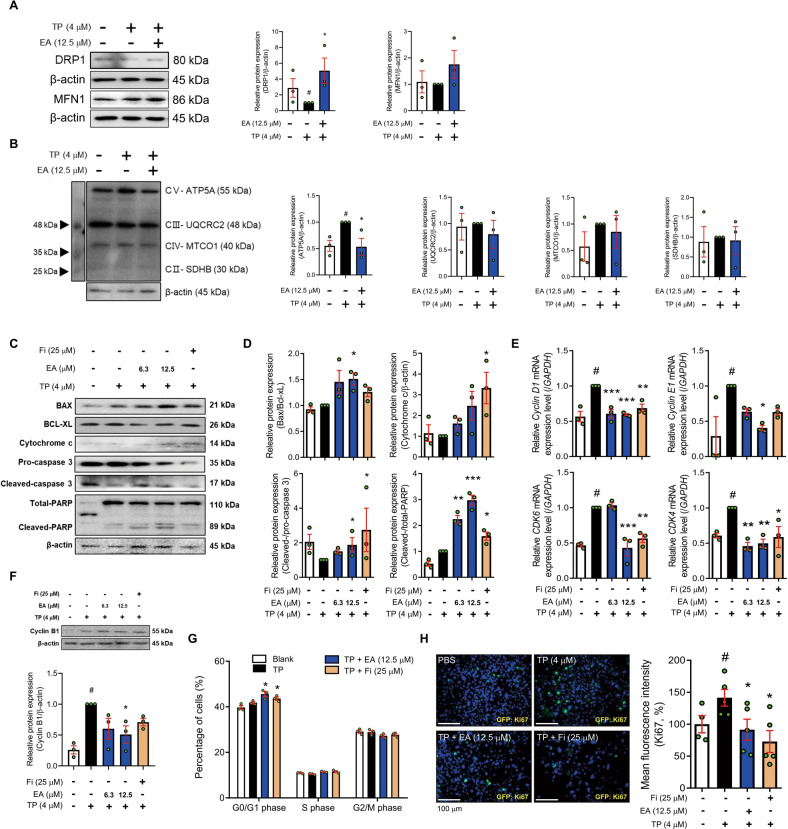
EA induces mitochondria-mediated apoptosis in TP-treated RWPE-1 cells. **A**, **B** Protein levels of DRP1, MFN1, and OXPHOS were measured by Western blot analysis. **C**, **D** Protein levels of Bax, Bcl_xL, cytochrome c, pro-/cleaved-caspase 3, and total/cleaved-PARP were analyzed by Western blot analysis. **E** mRNA expressions of *Cyclin D1, Cyclin E1, CDK4*, and *CDK6* were measured by RT-PCR, and data were normalized to *GAPDH*. **F** Protein level of cyclin B1 was measured by Western blot analysis. **G** Population of cell cycle (G0/G1, S, and G2/M) was analyzed using flow cytometry. **H** Protein level of Ki67 was detected using immunofluorescence staining (magnification 200×, scale bar 100 μm). β-actin was used as a loading control. All data are expressed as the mean ± S.E.M. of data from three or more separate experiments. Statistical differences were calculated by one-way ANOVA with post-hoc Tukey’s test. ^#^*p* < 0.05 vs. PBS-treated RWPE-1 cells, ^*^p < 0.05 and ^**^p < 0.01 vs. TP-treated RWPE-1 cells. PBS phosphate-buffered saline, TP testosterone propionate, EA ellagic acid, Fi finasteride.

The apoptotic system is known to be promoted by mitochondrial dysfunction associated with mitochondrial dynamics as well as cell cycle arrest, Bcl-2 family proteins, and OXPHOS complexes [16]. We also investigated whether EA regulates the expressions of apoptosis-related factors (Bcl-2-associated X protein: Bax, B-cell lymphoma-extra large (Bcl-xL), cytochrome c, caspase 9, and caspase 3), and OXPHOS complexes (ATP5A, UQCRC2, MTCO1, and SDHB), as well as mitochondrial dynamics-related factors (DRP1 and MFN1) in the TP-treated BPH rats. EA treatment increased the protein levels of DRP1 and MFN1 in the prostate of TP-treated BPH rats while the levels of ATP5A, MTCO, and SDHB were decreased by EA in the tissue (Fig. 3A, B). In addition, as shown in Fig. 3C, D, EA or Fi treatment elevated the ratios of Bax/Bcl-xL, cleaved-caspase 9/pro-caspase 9, cleaved-caspase 3/pro-caspase 3, and cleaved-PARP/total-PARP as well as cytochrome c in the prostate. Furthermore, EA or Fi treatment significantly decreased the levels of cyclin B1 and PCNA in the prostate (Fig. 3E). These were confirmed in TP-treated RWPE-1 cells (Fig. 4A–D).

Next, to investigate whether the expressions of cyclins and cyclin-dependent kinases (CDKs) were affected by EA treatment, we treated with EA (6.3 and 12.5 μM), and analyzed the mRNA expression of *Cyclin D1, Cyclin E1, CDK6*, and *CDK4* in TP-treated RWPE-1 cells (Fig. 4E). The treatment of EA down-regulated the mRNA expressions of cyclins and CDKs and the protein levels of cyclin B1 compared to TP treatment (Fig. 4F). From cell cycle analysis using flow cytometry, we confirmed that the ratio of G0/G1 phase-located cells in the population of the cell cycle (G0/G1, S, and G2/M) was increased by EA or Fi treatment (Fig. 4G). Furthermore, EA as well as Fi decreased the protein expression of Ki67 in the TP-treated RWPE-1 cells (Fig. 4H).

### EA reduces STAT3/NF-kB signaling axis in a TP-induced BPH rat model and RWPE-1 cells

**Fig. 5:**
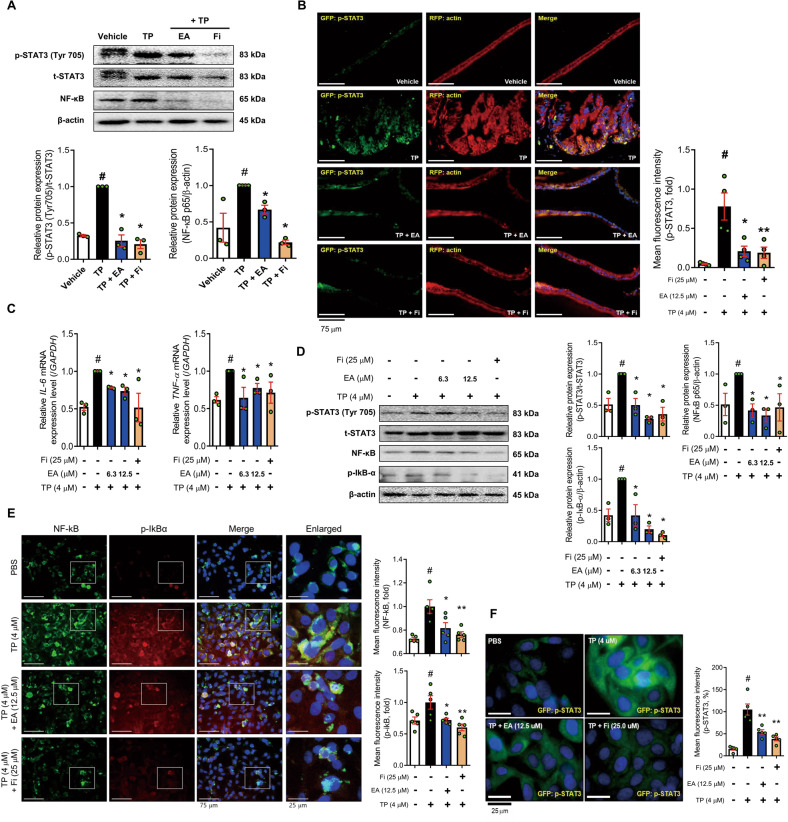
EA suppresses IL-6/STAT3/NF-kB axis in TP-induced BPH SD rats and TP-treated RWPE-1 cells. **A** Protein levels of p-STAT3 and NF-κB p65 were analyzed by Western blot analysis. β-actin was used as a loading control. The levels of protein were quantified using ImageJ software. **B** p-STAT3 (green), actin (red), and nuclei (blue) were detected in prostate tissue of rats by immunofluorescence staining (400× magnification, scale bar 75 μm). **C** mRNA expressions of *IL-6 and TNF*α were measured by RT-PCR, and data were normalized to *GAPDH*. **D** Protein levels of p-STAT3, t-STAT3, NF-κB p65, and p-IκBα were analyzed by Western blot analysis. **E** NF-κB (green), p-IκBα (red), and nuclei (blue) were detected using immunofluorescence staining (magnification 400×, scale bar 75 μm). **F** Intracellular p-STAT3 (green) and nuclei (blue) were detected by immunofluorescence staining (magnification 1000×, scale bar 25 μm). β-actin was used as a loading control. The levels of protein were quantified using ImageJ software. All data are expressed as the mean ± S.E.M. of data from three or more separate experiments. Statistical differences were evaluated using the unpaired *t*-test and a subsequent post-hoc one-tailed Mann–Whitney *U*-test. ^#^*p* < 0.05 vs. vehicle group or PBS-treated RWPE-1 cells, ^*^p < 0.05 vs. TP group or TP-treated RWPE-1 cells. PBS phosphate-buffered saline, TP testosterone propionate, EA ellagic acid, Fi finasteride.

The phosphorylated STAT3 and NF-κB levels were increased by TP treatment in the prostate. EA or Fi treatment reduced the levels of these proteins (Fig. 5A). Next, we conducted immunofluorescence staining and confirmed that the green fluorescence intensity of phospho-STAT3 was extensively detected in the prostate epithelial region isolated by actin staining. However, the expression of phospho-STAT3 in the EA or Fi treated group was decreased to a level similar to that of the vehicle group (Fig. 5B).

The exposure of RWPE-1 cells to TP increased the mRNA expression levels of pro-inflammatory cytokines (IL-6 and TNF-α), but its expression was decreased by EA or Fi treatment (Fig. 5C). Next, to investigate how EA affects the STAT3/NF-κB signaling axis, we confirmed the protein levels of STAT3, NF-κB, and p-nuclear factor of kappa light polypeptide gene enhancer in B-cell inhibitor alpha (p-IκBα) using immunofluorescence staining and Western blot in TP-treated RWPE-1 cells. The TP treatment greatly increased protein levels of p-STAT3, NF-κB, and p-IκBα compared to those in PBS-treated RWPE-1 cells. As expected, EA regulated the STAT3/NF-κB signaling axis by reducing pSTAT3, NF-κB, and p-IκBα levels. (Fig. 5D–F).

### EA alleviates androgen-mediated prostate hyperplasia through inhibition of STAT3 phosphorylation

**Fig. 6:**
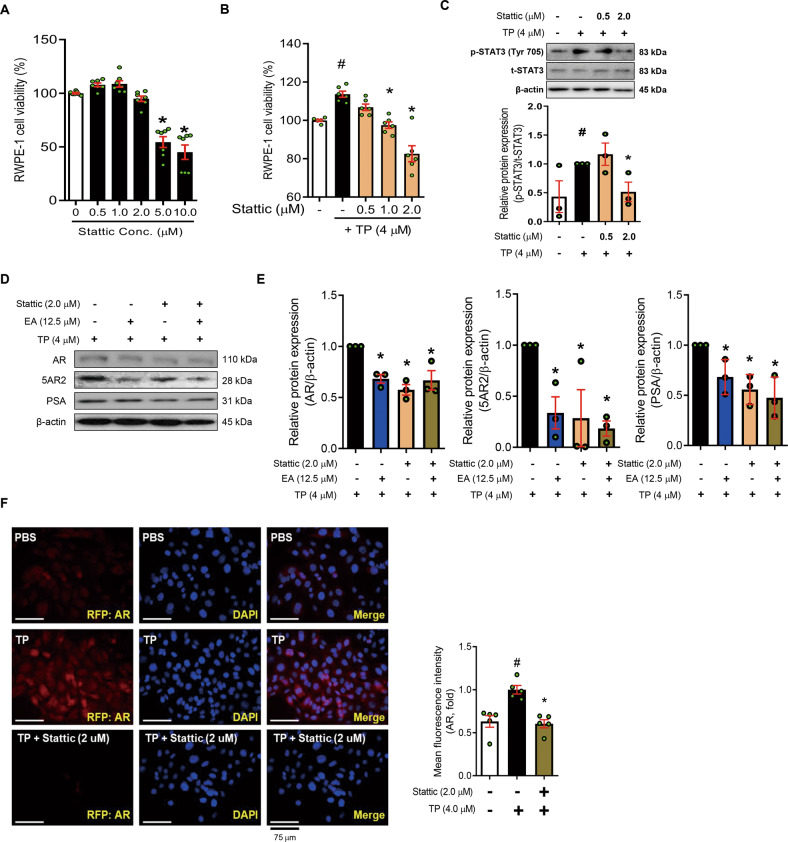
EA and Stattic suppresses AR/5AR2 by inhibiting of STAT3 in TP-treated RWPE-1 cells. **A**, **B** Cytotoxicity of Stattic on non-treated or TP (4.0 μM)-treated RWPE-1 cells was measured by WST-1 assay. **C** Phosphorylation level of STAT3 was analyzed in PBS or Stattic (0.5 and 2.0 μM) treated RWPE-1 cells by Western blot analysis and normalized to t-STAT3. **D**, **E** Protein levels of AR, 5AR2, and PSA were analyzed by Western blot analysis. β-actin was used as a loading control. **F** Protein level of AR was detected using immunofluorescence staining. The levels of protein were quantified using ImageJ software. All data are expressed as the mean ± S.E.M. of data from three or more separate experiments. Statistical differences were evaluated using the unpaired *t*-test and a subsequent post-hoc one-tailed Mann–Whitney *U*-test. ^#^*p* < 0.05 vs. PBS-treated RWPE-1 cells, ^*^p < 0.05 vs. TP-treated RWPE-1 cells. PBS phosphate-buffered saline, TP testosterone propionate, EA ellagic acid.

Because we found that EA inhibits the p-STAT3 level while TP treatment increases the STAT3 phosphorylation, we next confirmed whether the inhibition of STAT3 alleviates the proliferation of TP-treated RWPE-1 cells. As shown in Fig. 6A–C, Stattic, a potent STAT3 inhibitor, does not affect the viability of RWPE-1 cells at concentrations of <2.0 μM, and effectively reduces cell proliferation and STAT3 phosphorylation in TP-treated RWPE-1 cells. To investigate whether the inhibition of STAT3 phosphorylation also affects the AR signaling axis, we dosed TP-treated RWPE-1 cells with Stattic or EA and confirmed the protein levels of AR, 5AR2, and PSA using Western blot. The protein levels of AR, 5AR, and PSA in TP-treated RWPE-1 cells were reduced by Stattic or EA treatment (Fig. 6D, E). Furthermore, immunofluorescence staining showed that Stattic downregulates the AR expression in TP-treated RWPE-1 cells (Fig. 6F).

## Discussion

During the process of BPH development, an imbalance between testosterone and DHT is a major pathophysiological factor in prostate remodeling [17]. DHT initiates AR signaling pathways through a combination with AR in androgen binding sites [18]. Molecules of a DHT/AR complex move into a nucleus and then bind to the ARE present in the promoters of target genes leading growth and survival of prostate epithelial cells [19]. Next, they trigger the proliferation of prostate cells by activation of cell cycle progression and increase PSA expression levels in the cells [20]. Increased expressions of AR, 5AR2, and PSA correlate with prostate hyperplasia, and these markers are increased markedly in patients and in animal models with BPH [21, 22]. The experimental BPH animal models induced by chronic injection of TP have been widely used to confirm etiological and pathophysiological evidence as well as pharmacological evidence for BPH [23, 24]. The present study used the TP-induced BPH rat model to investigate the therapeutic effect of EA and we found that EA significantly reduced the prostate size, epithelial thickness, serum DHT levels, and the protein levels of AR, 5AR2, and PSA in the TP-induced BPH rat model.

In a normal prostate, a balance between apoptosis and proliferation of cells is achieved to control excessive proliferation of prostate epithelial cells, while microenvironmental changes such as increasing DHT levels lead to excessive proliferation due to suppression of cell death, causing prostate hyperplasia [6]. Therefore, previous studies have confirmed whether candidates for BPH treatment, in addition to the hormonal factors, play a role in regulating Bcl-2 family proteins and caspases [25]. In the present study, EA treatment significantly induces mitochondrial-mediated apoptosis as well as inhibits DHT/AR signaling pathways in TP-induced BPH rats and TP-treated RWPE-1 cells.

BPH is an age-related disease characterized by the growth of epithelial and stromal cells, but also includes a microenvironment of chronic inflammation [26]. The inflammatory sign can be induced not only by viral or bacterial antigens but also metabolic disorders such as hormone imbalance. The cause of prostate chronic inflammation during prostate hyperplasia is still unclear, but it has been confirmed that inflammatory cells such as T lymphocytes and macrophages infiltrate the prostates of BPH patients [27]. Emerging data have shown that pro-inflammatory cytokines, including IL-6 and IL-8 secreted by these inflammatory cells, selectively promote the proliferation of prostate epithelial cells [26, 28]. Other studies have confirmed that epithelial and stromal cells, as well as infiltrated immune cells, have cytokine receptors on their membrane surface and can participate in local prostate inflammatory microenvironments [29]. Our findings showed that TP increased pro-inflammatory mediators such as IL-6 and TNF-α in prostate tissue and RWPE-1 prostate epithelial cells. Moreover, EA significantly reduced the expression of IL-6 and TNF-α through inhibition of the STAT3/NF-kB signaling axis in the prostate of BPH rats and TP-treated RWPE-1 cells.

STAT3 and NF-kB act as major transcription factors in many cellular processes, such as cell growth, apoptosis, and inflammation [30]. The activation of these factors is induced by pro-inflammatory mediators, in particular IL-6, and is known to promote prostate carcinogenesis as well as the development and progression of BPH [31]. The increased IL-6 expression by activation of ikBα and NF-kB in the BPH microenvironment leads to phosphorylation of STAT3 [32]. The phosphorylated STAT3 undergoes a process of dimerization for stabilization and moves to the nucleus. Then, it initiates transcription of pro-inflammatory genes such as IL-6, TNF-α, and IL-8 [33]. The released pro-inflammatory molecules might aggravate the BPH by inflammation reaction via autocrine/paracrine systems [34]. Therefore, inhibition of phosphorylation of STAT3 is expected to play a role as a negative regulator in the development and progression of BPH [35]. Although further investigation is required for the detailed roles of STAT3 in the mechanics of either BPH development or progression, Stattic, a potent inhibitor of STAT3, as well as EA markedly reduced not only cell proliferation but also the protein levels of AR, 5AR2, and PSA in TP-treated RWPE-1 cells.

In conclusion, EA improves androgen-mediated prostate hyperplasia induced by AR signaling and STAT3 activation in the TP-induced BPH animal and cell models. These findings advance understanding of the pharmacological role of EA in the treatment of BPH.

## Experimental procedures

### Chemical reagents

EA (PubChem CID: 5281855) was purchased from Sigma Chemicals (St. Louis, MO, USA) and dissolved in distilled water (DW). Testosterone propionate (TP, PubChem CID: 5995) was provided from Wako pure chemical industries (Osaka, Japan), and finasteride (≥ 98% pure, PubChem CID: 57363) was purchased from Sigma-Aldrich Inc. (Tokyo Chemical Industry Co., Ltd., Japan). The antibodies used in this study were shown in Supplementary Table 1.

### Animal experiments

Six-week-old male Sprague-Dawley (SD) rats were purchased from the Dae-Han Experimental Animal Center (Dae-Han Biolink, Eumsung, Korea) and kept for 1 week prior to the experiments. The animals were all maintained under conditions in accordance with the regulation issued by the Institutional Review Board of Kyung Hee University (confirmation number KHUASP(SE)-19-406).

BPH was induced by subcutaneous injection of TP (5 mg/kg) dissolved in corn oil containing 5% ethanol for 8 weeks, as described previously [36]. In detail, the rats (*n* = 24) were randomly divided into two groups, either the vehicle group (*n* = 6) that were subcutaneously injected with corn oil containing 5% ethanol, or the TP group (*n* = 18) that were subcutaneously injected with TP. Next, after 4 weeks of injection, the rats of the TP group were randomly divided into three groups (*n* = 6 per group). Group A included members of the TP group that were subcutaneously injected with TP. Group B included members of the TP + EA group that were subcutaneously injected with EA (20 mg/kg) and TP. Group C included members of the TP + finasteride (TP + Fi) group, a positive control group, into which Fi (1 mg/kg) and TP were subcutaneously injected. All groups were supplemented daily in the inguinal region of the rats. After 4 weeks, the rats (*n* = 24) were sacrificed by cervical dislocation under CO_2_ asphyxiation. All analyses were conducted in investigator blinded fashion.

### Hematoxylin and eosin staining

Hematoxylin-eosin (H&E) staining was performed as previously reported [37]. Microscopic examinations were performed, and photographs were taken under a regular light microscope. The prostate epithelial thickness was calculated using the ImageJ software program (National Institute of Health, Bethesda, MD, USA).

### Cell culture

RWPE-1, a normal human prostate epithelial cell line, was purchased from the American Type Culture Collection (Manassas, VA, United States). The cells were cultured in Roswell Park Memorial Institute (RPMI) 1640 media supplemented with 10% FBS, 100 U/mL of penicillin, and streptomycin in a CO_2_ incubator at 37 °C with 5% CO_2_.

### Cell viability assay

To measure cell viability, we used the WST-1 assay. The WST-1 assay was performed as previously reported [37]. The absorbance was measured using a VERSAmax microplate reader (Molecular Devices, Sunnyvale, CA, United States) at 450 nm.

### Western blot analysis

Protein expression analysis was performed as previously reported [37]. The membranes were blocked in 5% skim milk and incubated with the respective primary antibody (1:1000) overnight at 4 °C after incubation with an HRP-conjugated secondary antibody (1:10000) for 1 h at room temperature. The proteins were visualized using the ECL advance kit.

### Immunofluorescence assay

An immunofluorescence assay was performed as previously reported [38]. The fluorescence was detected using an EVOS Cell Imaging System (Thermo Scientific, Carlsbad, CA, USA).

### MDA measurement

MDA contents were detected using the EZ-lipid peroxidation (TBARS) assay kit (DoGen Bio, Seoul, Korea). These levels were confirmed based on the manufacturer’s instructions.

### Catalase activity measurement

Catalase activity was detected using the EZ-catalase assay kit (DoGen Bio, Seoul, Korea). These levels were confirmed based on the manufacturer’s instructions.

### RNA isolation and real-time reverse transcription-polymerase chain reaction

RNA isolation and real-time RT-PCR were performed as previously described [38]. The relative gene expressions were calculated based on the comparative CT method using StepOne software v2.1 (Applied Biosystems, Foster City, CA, USA). The mRNA expression of *GAPDH* was used as an endogenous control. The primers used in this study were shown in Supplementary Table 2.

### Cell cycle analysis by flow cytometry

RWPE-1 cells were seeded at a density of 8 × 10^5^ cells/well in 6-well plates. After 12 h, the cells were exposed to TP (4 μM) for 1 h. Then, they were treated with EA (12.5 and 25.0 μM) or Fi (25 μM) for 24 h. The cells were washed in 1% FBS in phosphate buffered-saline (PBS) and fixed using 500 μL of 70% ethanol overnight at −20 °C. After washing, the cell pellet was left in PI staining solution at room temperature for 30 min in the dark. The stained cells were analyzed by flow cytometry.

### Statistical analysis

All data are expressed as mean ± SEM of independent experiments. Statistical differences were evaluated using the Student’s *t*-test or one-way ANOVA, and a subsequent *post-hoc* test via Prism 8 (GraphPad Software, San Diego, CA, USA). Values of *p < 0.05 and **p < 0.01 were considered statistically significant.

## Supplementary information

AJ Checklist

Supplymental figure and table

Original Data File (WB and IF)

## Data Availability

All data generated or analyzed during this study are available from the corresponding author on reasonable request.
