# Supplementary material for: Ellagic acid improves benign prostate hyperplasia by regulating androgen signaling and STAT3
Source: Cell Death Dis. 2022 Jun 17;13(6):554. doi: 10.1038/s41419-022-04995-3 (PMC9205887; doi:10.1038/s41419-022-04995-3)
Supplement: Supplementary file 2 — Supplymental figure and table [file 41419_2022_4995_MOESM2_ESM.pdf]

Supplementary information

Ellagic Acid Improves Benign Prostate Hyperplasia and Testicular Damage in Male Rats

Park et al.

Supplementary Figure 1. Effect of EA on the parameters of liver and kidney injury in serum of TP-induced BPH rats

Supplementary Table 1. List of primer sequences used for qPCR.

Supplementary Table 2. List of primary and secondary antibodies used in this study.

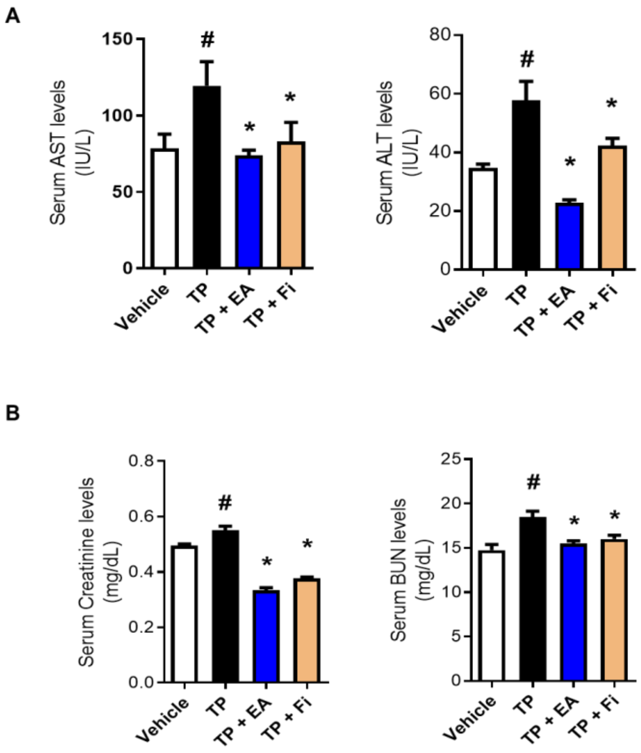

Supplementary Figure 1. Effect of EA on the parameters of liver and kidney injury in serum of TP-induced BPH rats.

(A) Serum levels of AST and ALT were measured. (B) Serum levels of creatinine and BUN were measured. All data are expressed as the mean ± S.E.M. of data from three or more separate experiments. $^{\#}p < 0.05$ vs. vehicle group, $^{*}p < 0.05$ vs. TP group. TP, testosterone propionate; EA, ellagic acid; Fi, finasteride; AST, asparate aminotransferase; ALT, alanine transaminase; BUN, blood urea nitrogen.

Supplementary Table 1. List of primer sequences used for qPCR.

Genes	Forward (5’ to 3’)	Reverse (5’ to 3’)
h-AR	CCTGTTCCGCAACTTACAC	GGACTTGTGACTGCGGTACTCA
h-5AR2	AACACGGCGCGATGCAGGTT	CCGTGTGCTTCCCGTAGCCG
h-IL6	ACAGCCACTCACCTCTTCAG	CCATCTTTTTCAGCCATCTT
h-TNFα	CCCGAGTGACAAGCCTGTAG	GATGGCAGAGAGGAGGTTGAC
h-GAPDH	CAAGGTCATCCATGACAACTTTG	GTCCACCACCTGTTGCTGTAG

Supplementary Table 2. List of primary and secondary antibodies used in this study.

**Category**	**Product**	**MFG. Co**	**Product number**
Western blot 1st Ab	AR	Thermo Fisher Scientific	PA1-111B
	PSA	Santa Cruz Biotechnology	sc-7316
	SOD1	Santa Cruz Biotechnology	sc-101523
	Catalase	Santa Cruz Biotechnology	sc-27180
	PCNA	Santa Cruz Biotechnology	sc-25280
	Cyclin B1	Santa Cruz Biotechnology	sc-752
	p-IkBα	Santa Cruz Biotechnology	sc-8404
	NF-kB p65	Santa Cruz Biotechnology	sc-372
	5AR2	Biorbyt	orb101414
	p-STAT3	Cell Signaling Technology	9145s
	t-STAT3	Cell Signaling Technology	9139s
	Bax	Cell Signaling Technology	2772s
	Bcl-xL	Cell Signaling Technology	2764s
	Cytochrome c	Cell Signaling Technology	4272s
	Caspase 9	Cell Signaling Technology	9508s
	Caspase 3	Cell Signaling Technology	9662s
	PARP	Cell Signaling Technology	9532s
	β-actin	Cell Signaling Technology	9700s
Western blot 2nd Ab	Goat anti-Mouse IgG	Thermo Fisher Scientific	G-21040
	Goat anti-Rabbit IgG	Thermo Fisher Scientific	G-21234
Immunofluorescence staining 1st Ab	p-STAT3	Cell Signaling Technology	9145s
	AR	Thermo Fisher Scientific	PA1-111B
	Actin	Santa Cruz Biotechnology	sc-8432
	p-IkBα	Santa Cruz Biotechnology	sc-8404
	NF-kB p65	Santa Cruz Biotechnology	sc-372
	Ki67	Abcam	ab15580
Immunofluorescence staining 2nd Ab	Alexa Fluor 488 Goat anti-rabbit	Thermo Fisher Scientific	A-11008
	Alexa Fluor 633 Goat anti-Mouse	Thermo Fisher Scientific	A-21052
