# Supplementary material for: Ellagic acid improves benign prostate hyperplasia by regulating androgen signaling and STAT3
Source: Cell Death Dis. 2022 Jun 17;13(6):554. doi: 10.1038/s41419-022-04995-3 (PMC9205887; doi:10.1038/s41419-022-04995-3)
Supplement: Supplementary file 3 — Original Data File (WB and IF) [file 41419_2022_4995_MOESM3_ESM.pdf]

Figure 1i

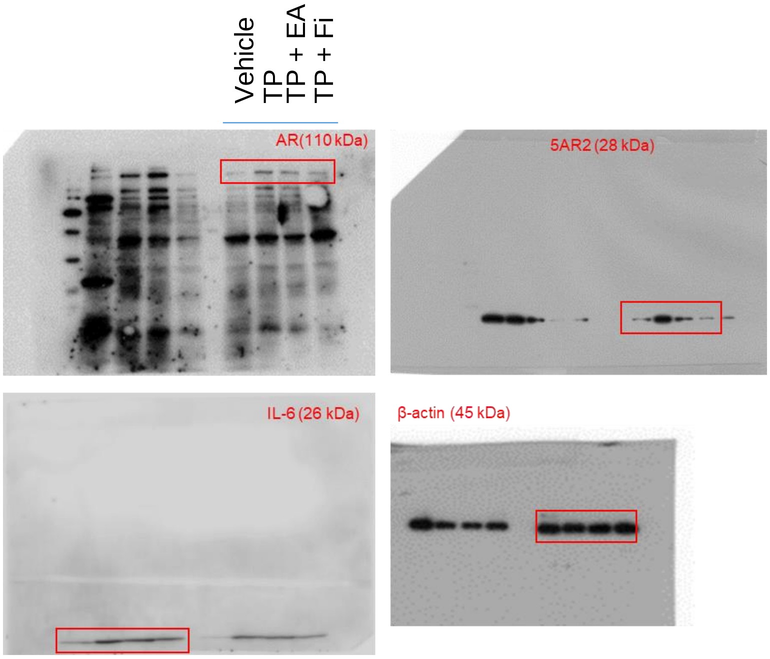

Figure 1k

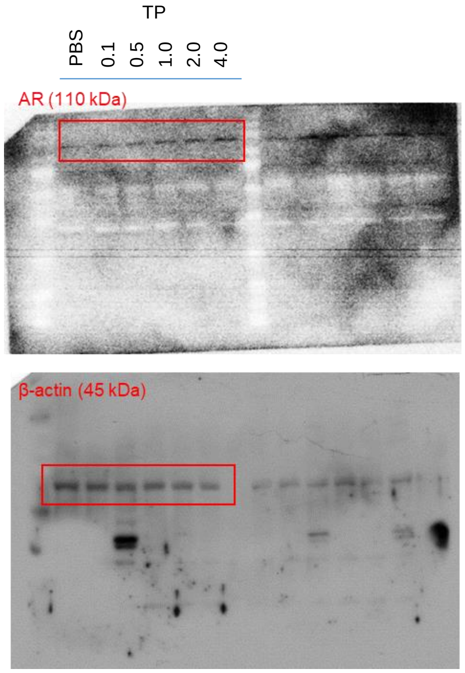

Figure 1o

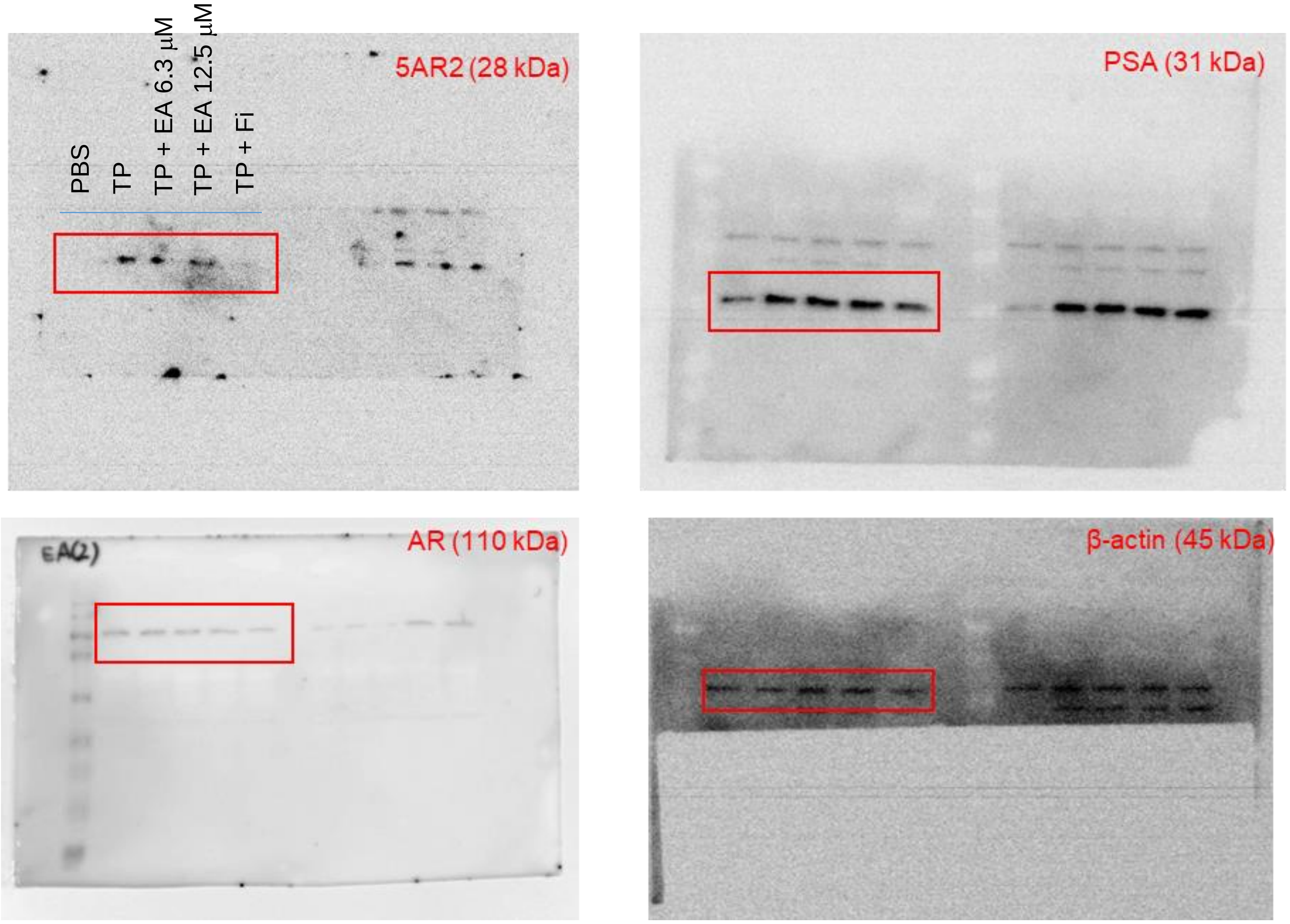

Figure 1p

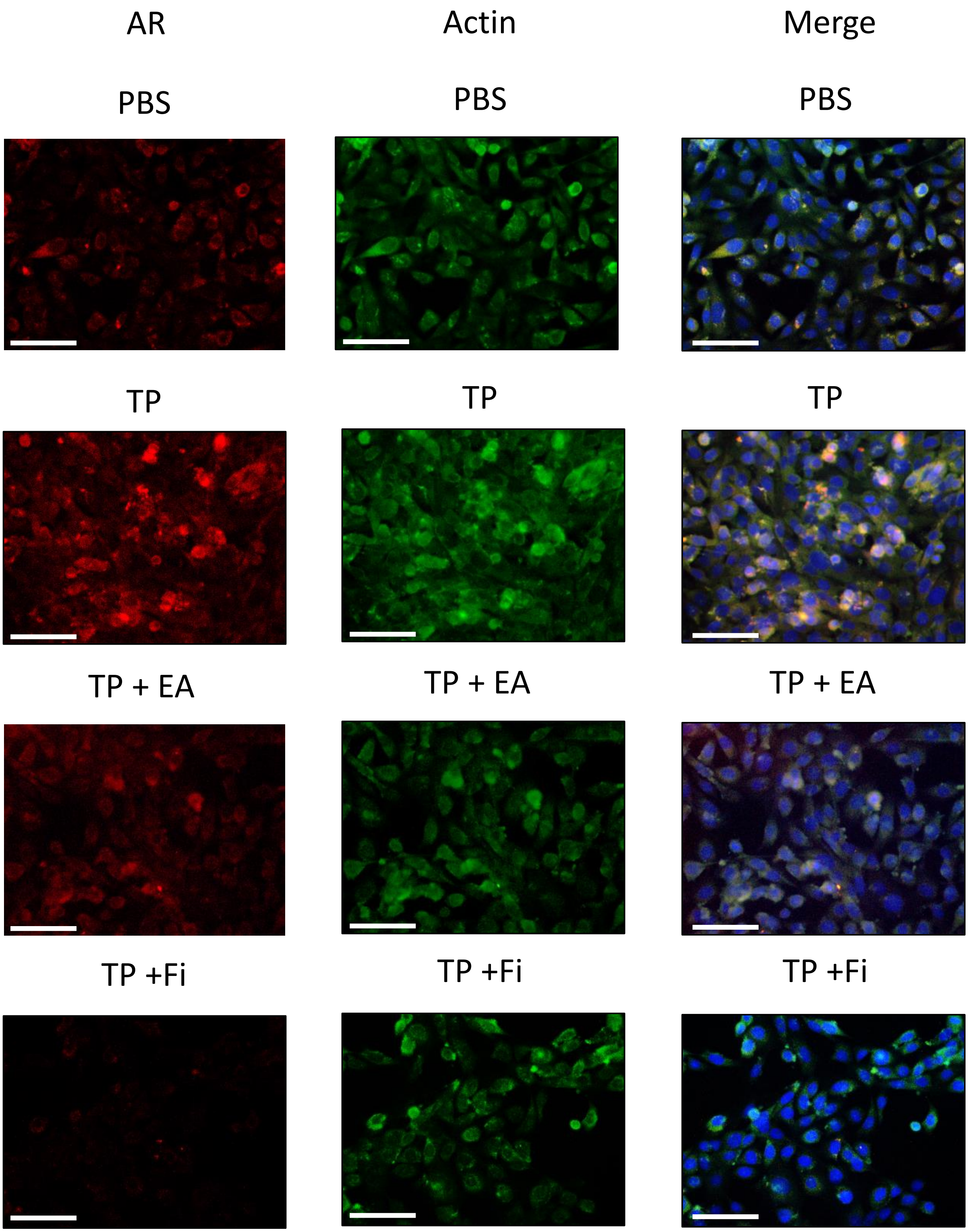

X400

Figure 2b

PCNA (33 kDa)

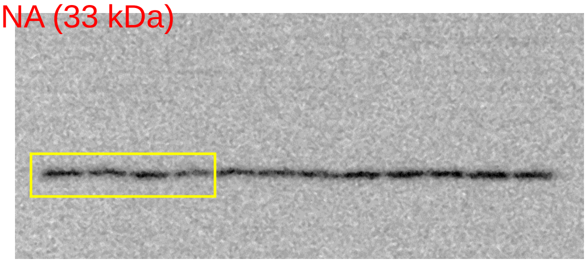

β-actin (45 kDa)

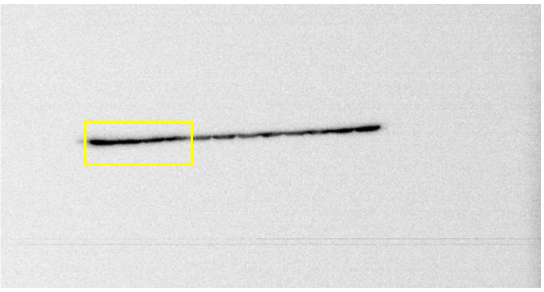

Vehicle
TP
TP + EA
TP + Fi

Caspase 3 (35, 20, 17 kDa)

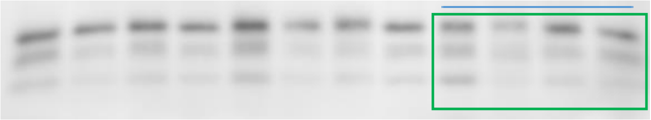

PCNA (33 kDa)

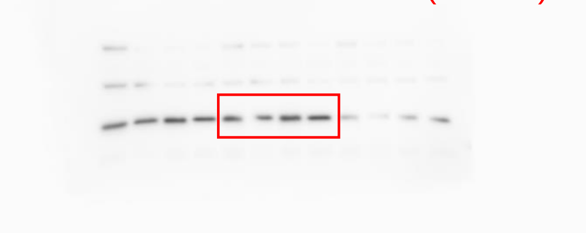

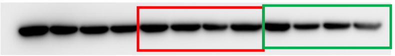

β-actin (45 kDa)

Figure 2e

Catalase (64 kDa)

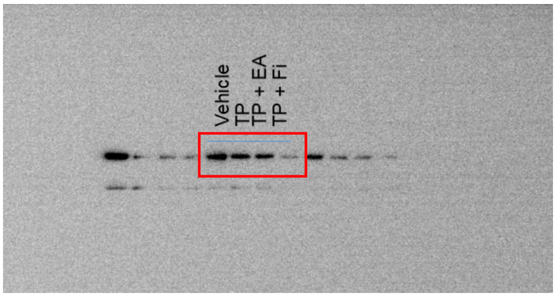

β-actin (45 kDa)

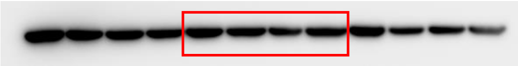

SOD1 (23 kDa)

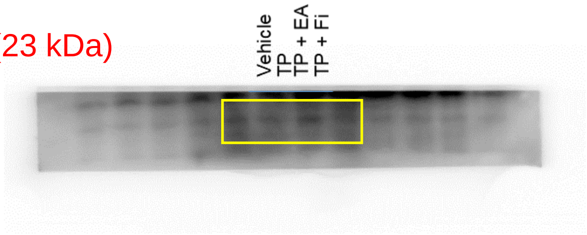

β-actin (45 kDa)

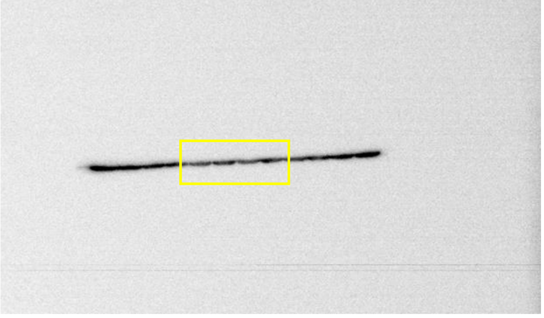

Figure 2h

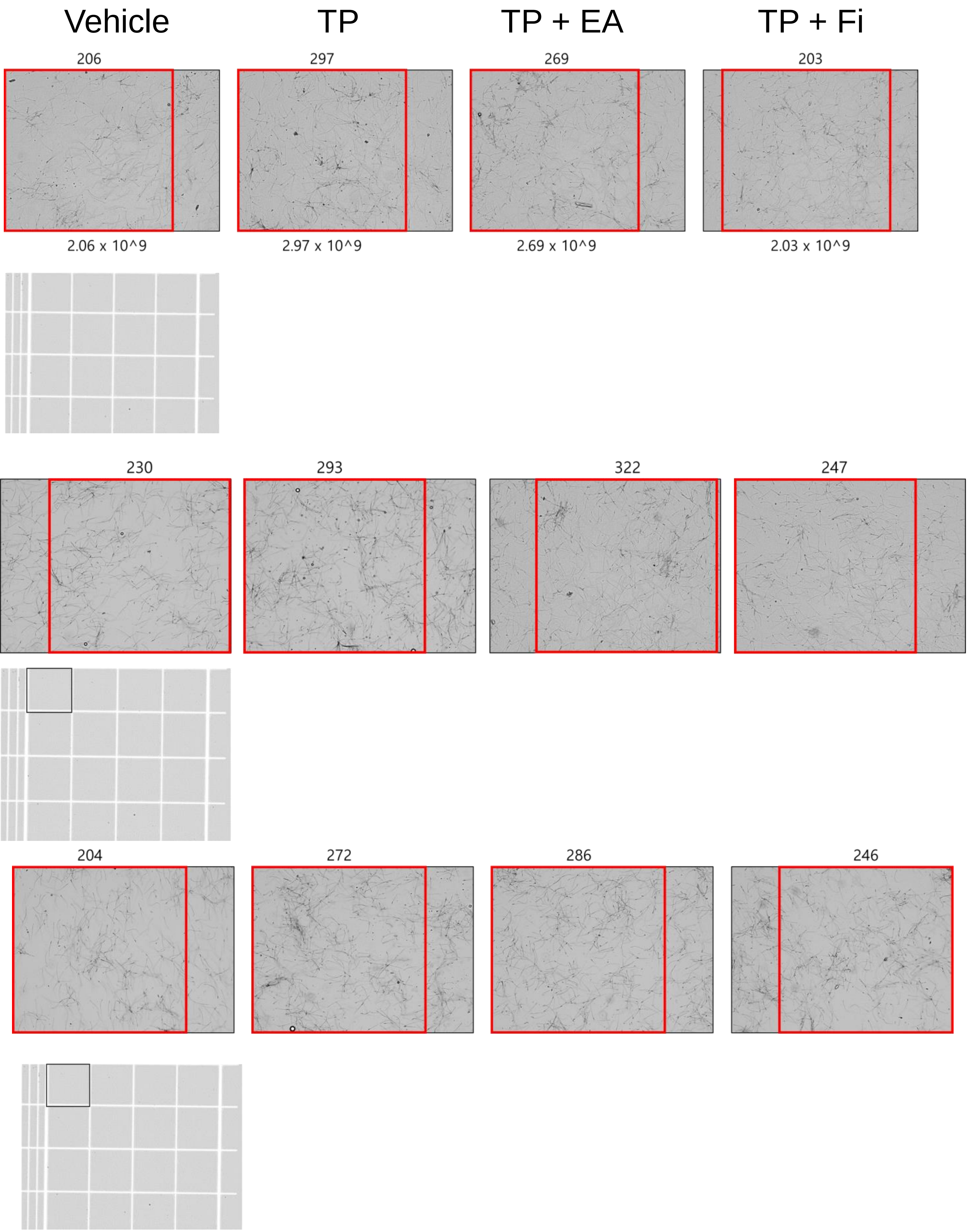
Vehicle
TP
TP + EA
TP + Fi
206
297
269
203
2.06 x 10^9
2.97 x 10^9
2.69 x 10^9
2.03 x 10^9
230
293
322
247
204
272
286
246

Figure 3a, b

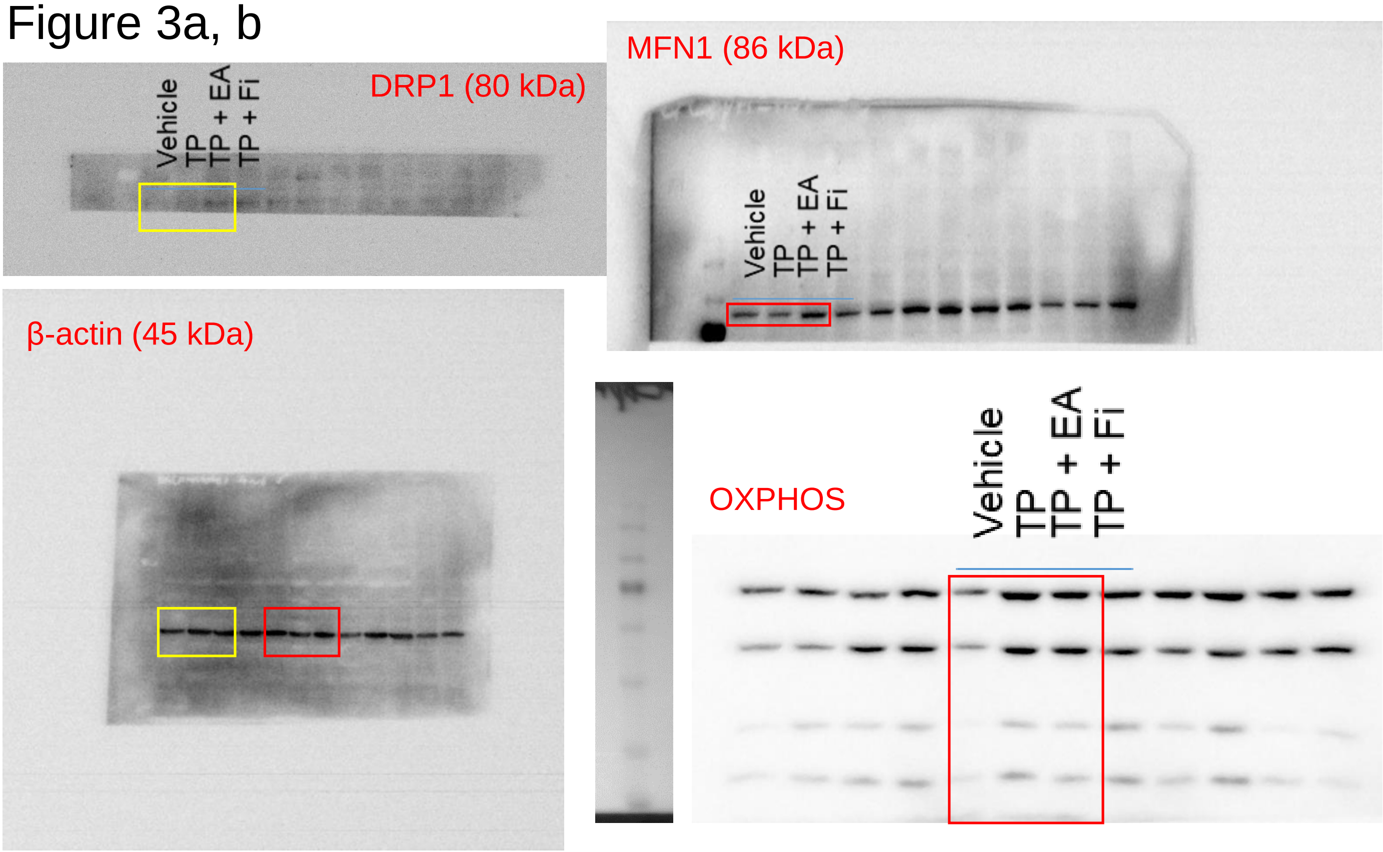

Figure 3c, e

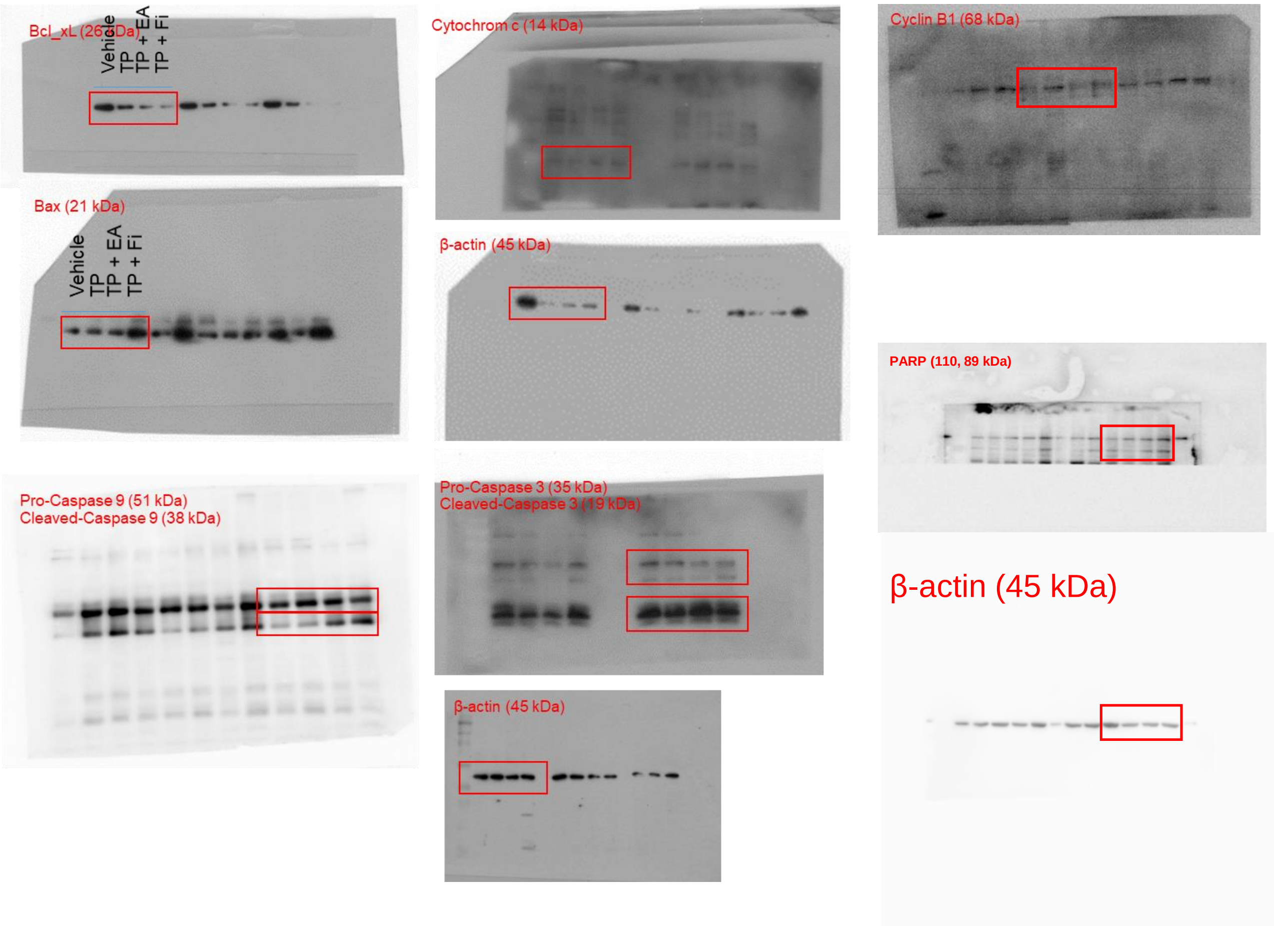

Figure 4a, b

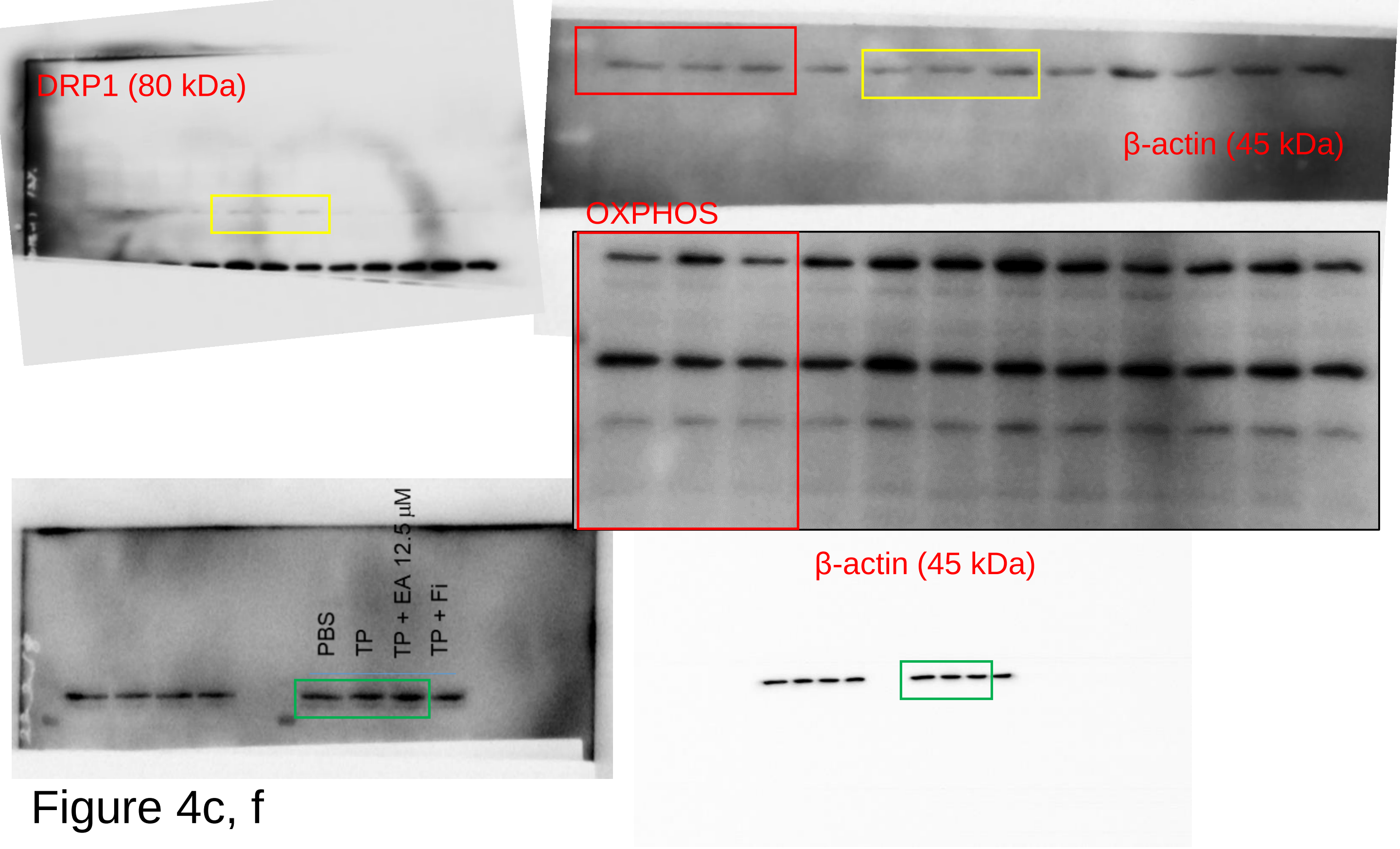

Figure 4c, f

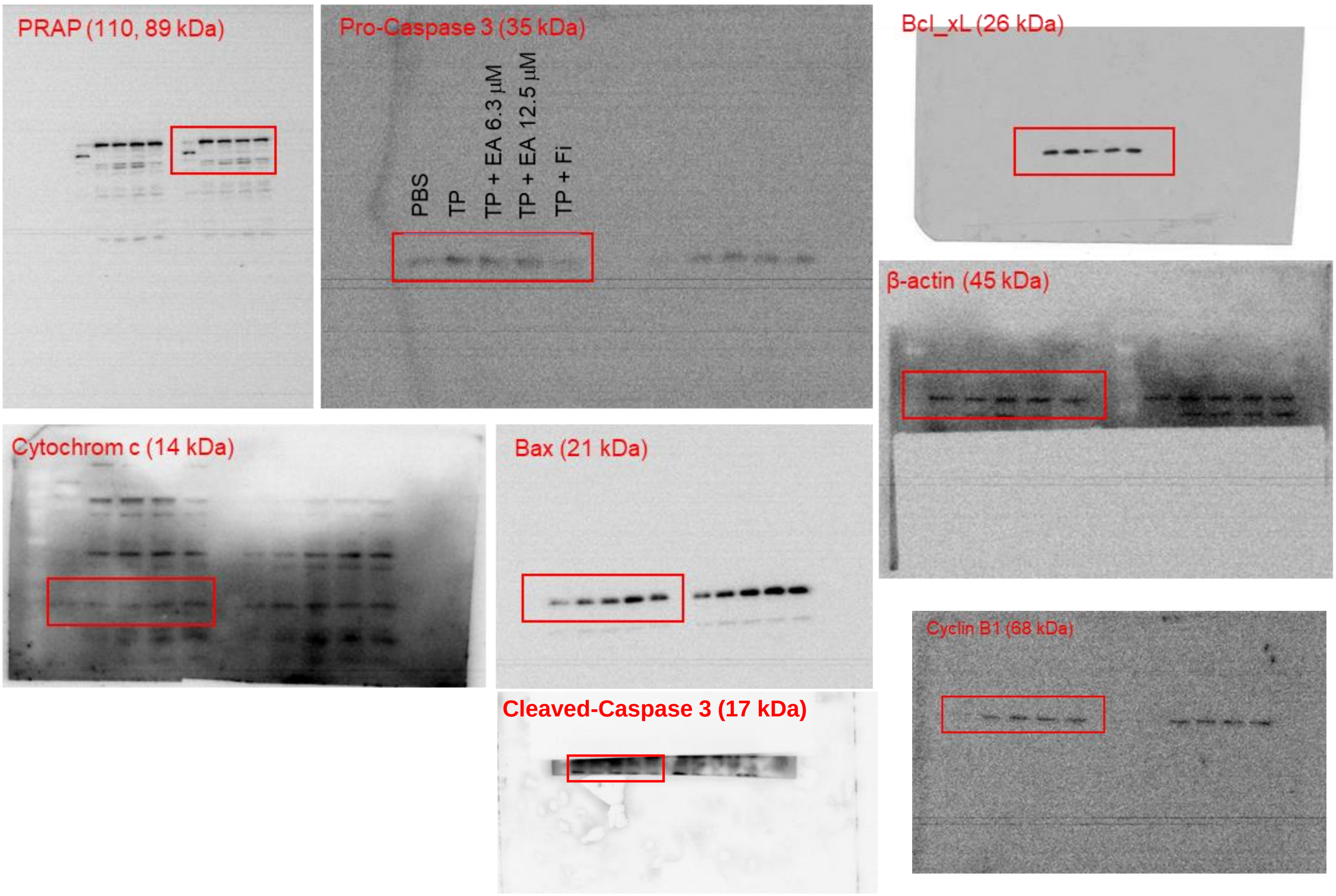

Figure 4h

Ki67/DAPI merge

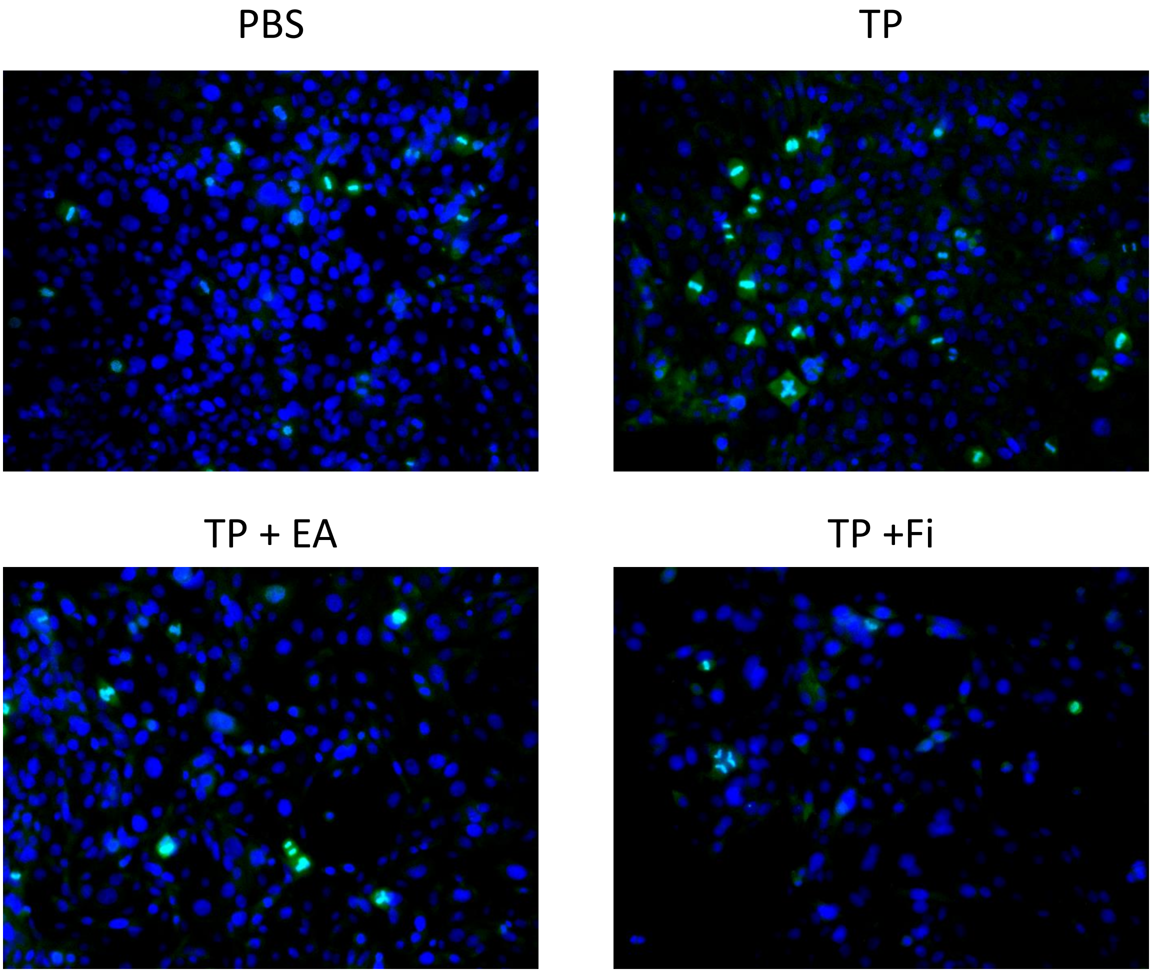

X200

Figure 5a

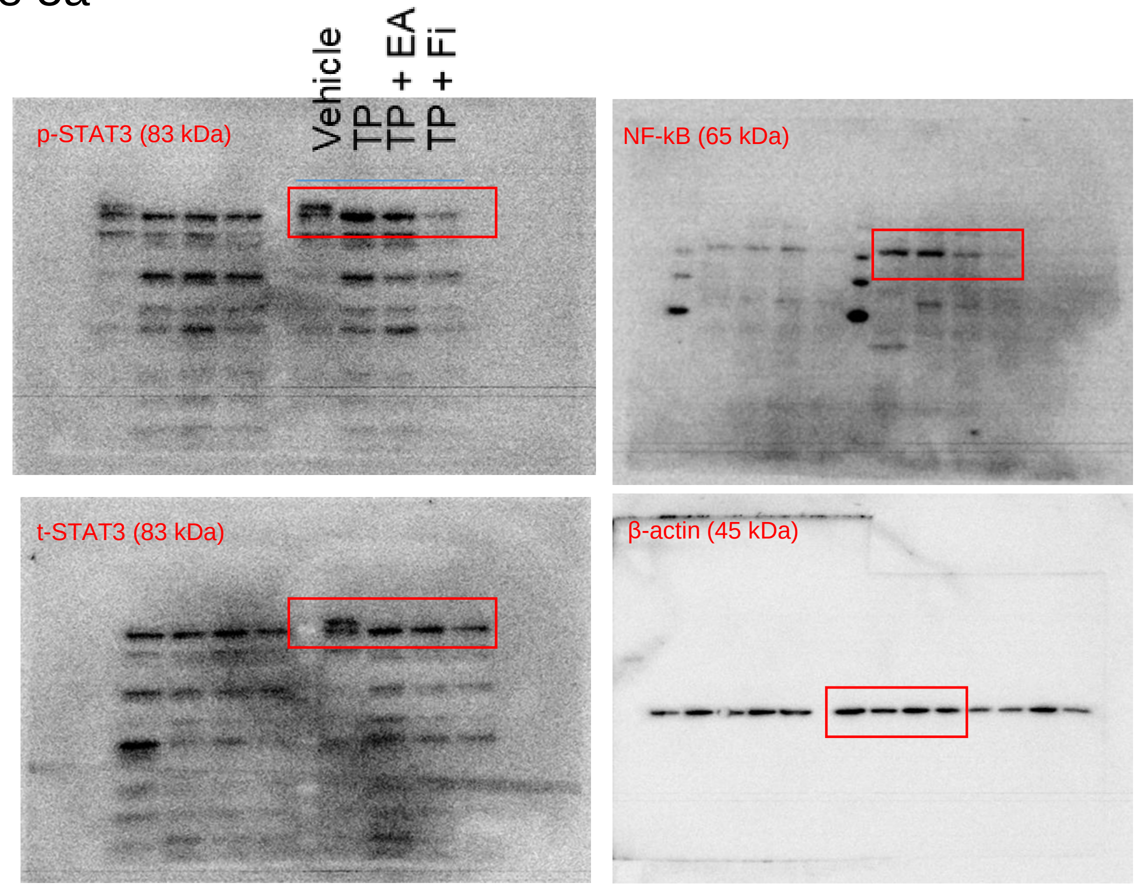

Figure 5d

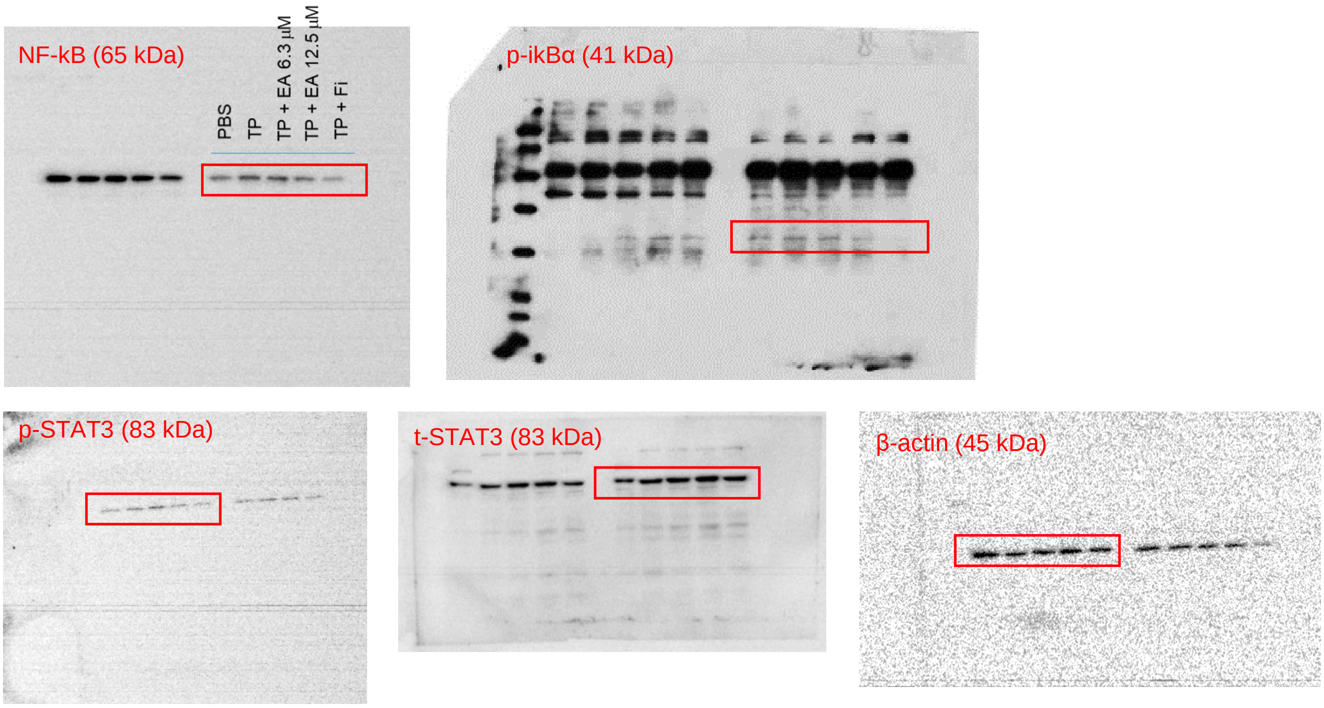

Figure 5b

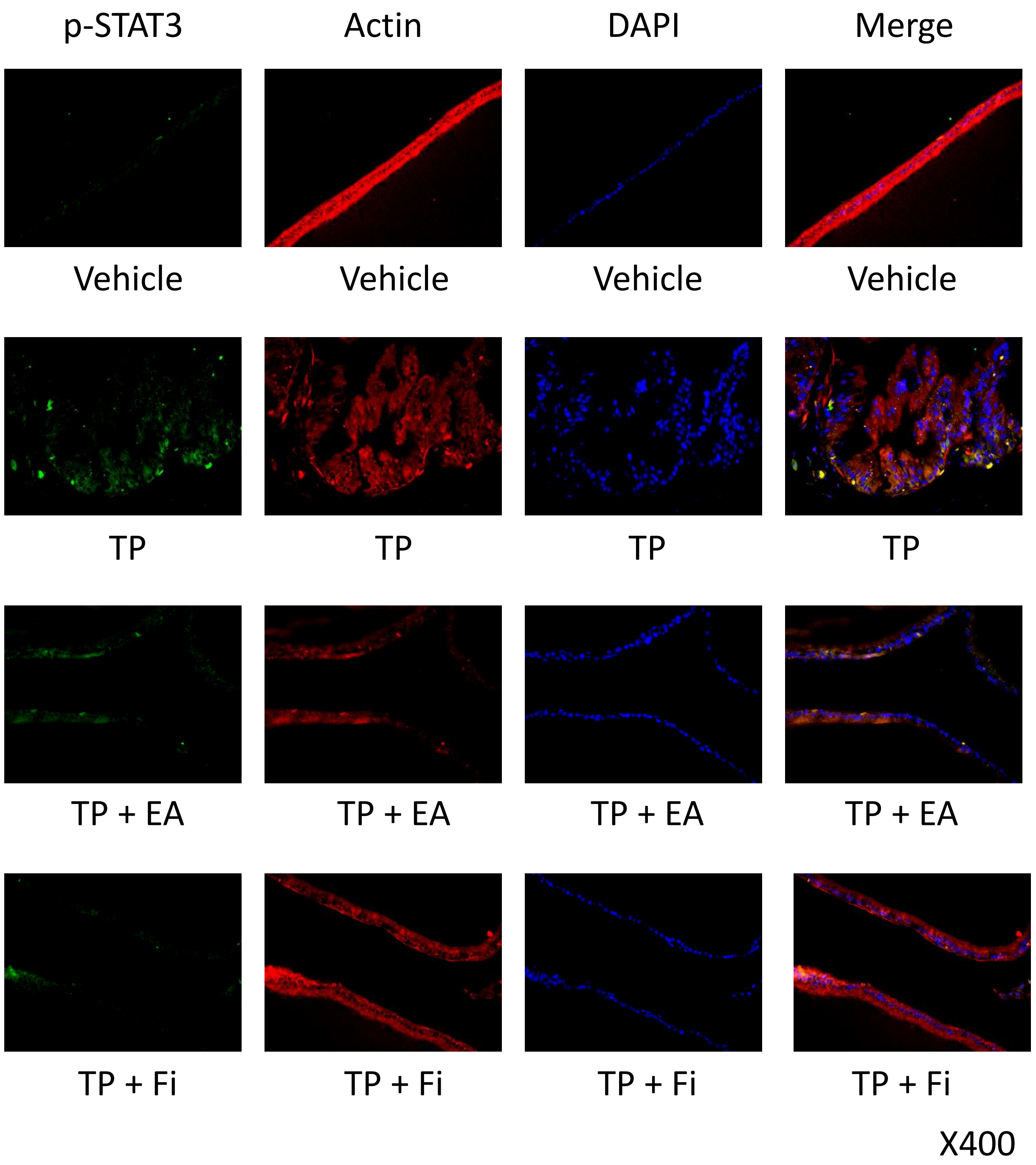

p-STAT3
Actin
DAPI
Merge
Vehicle
Vehicle
Vehicle
Vehicle
TP
TP
TP
TP
TP + EA
TP + EA
TP + EA
TP + EA
TP + Fi
TP + Fi
TP + Fi
TP + Fi
X400

Figure 5e

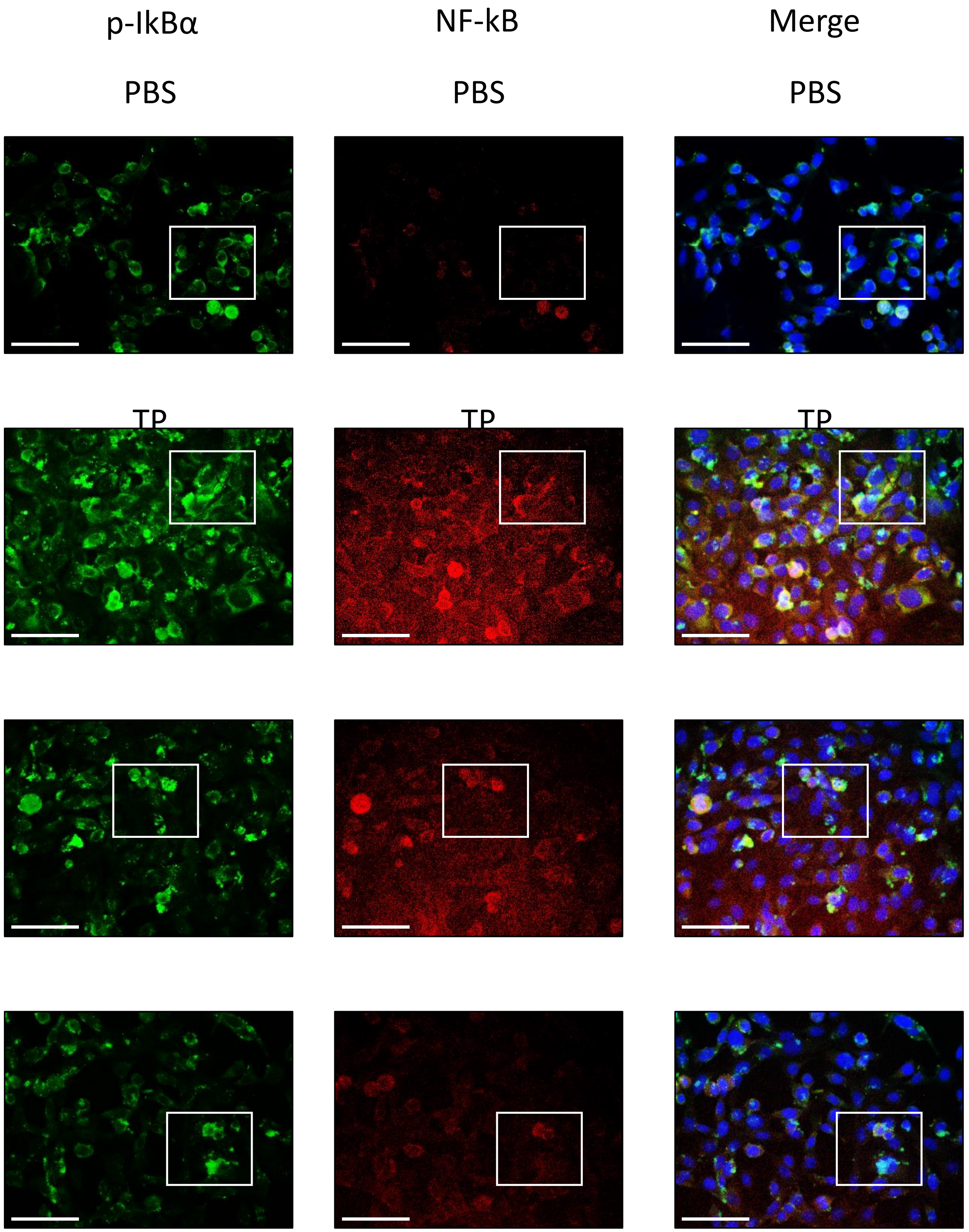

X400

Figure 5f

p-STAT3/DAPI merge

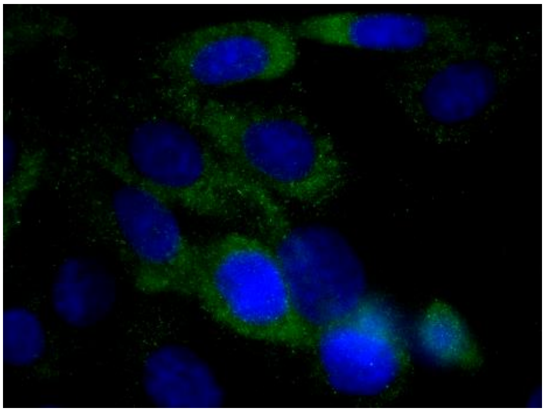

PBS

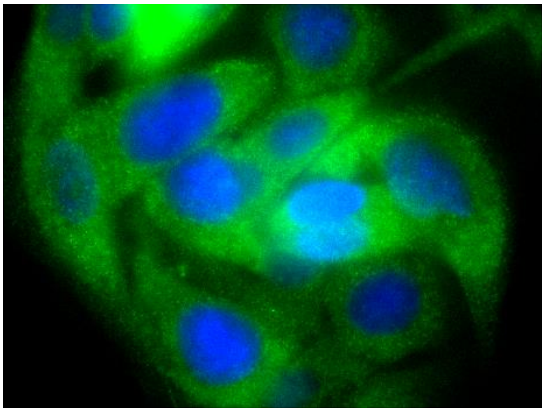

TP

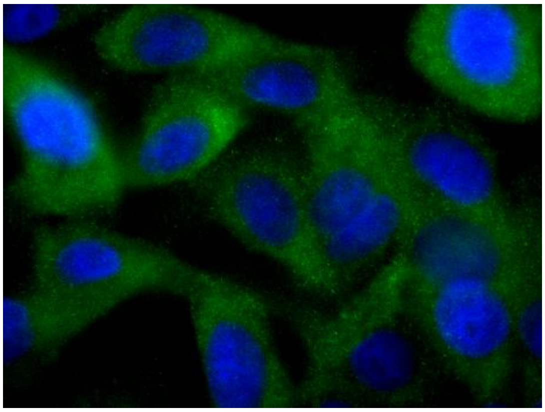

TP + EA

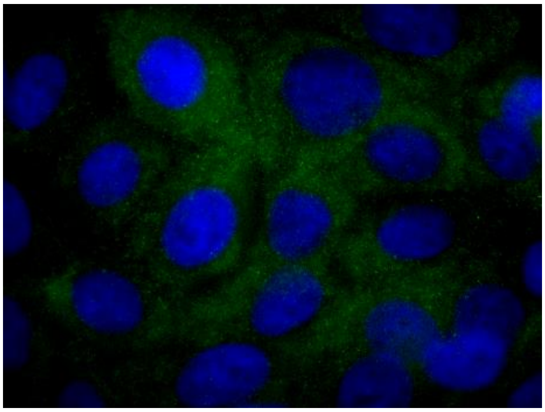

TP +Fi

X1000

Figure 6c

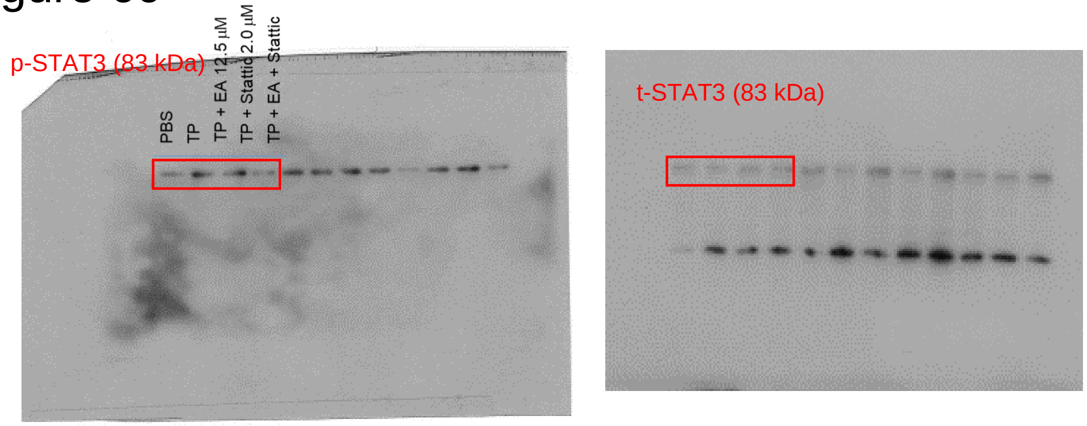

Figure 6d

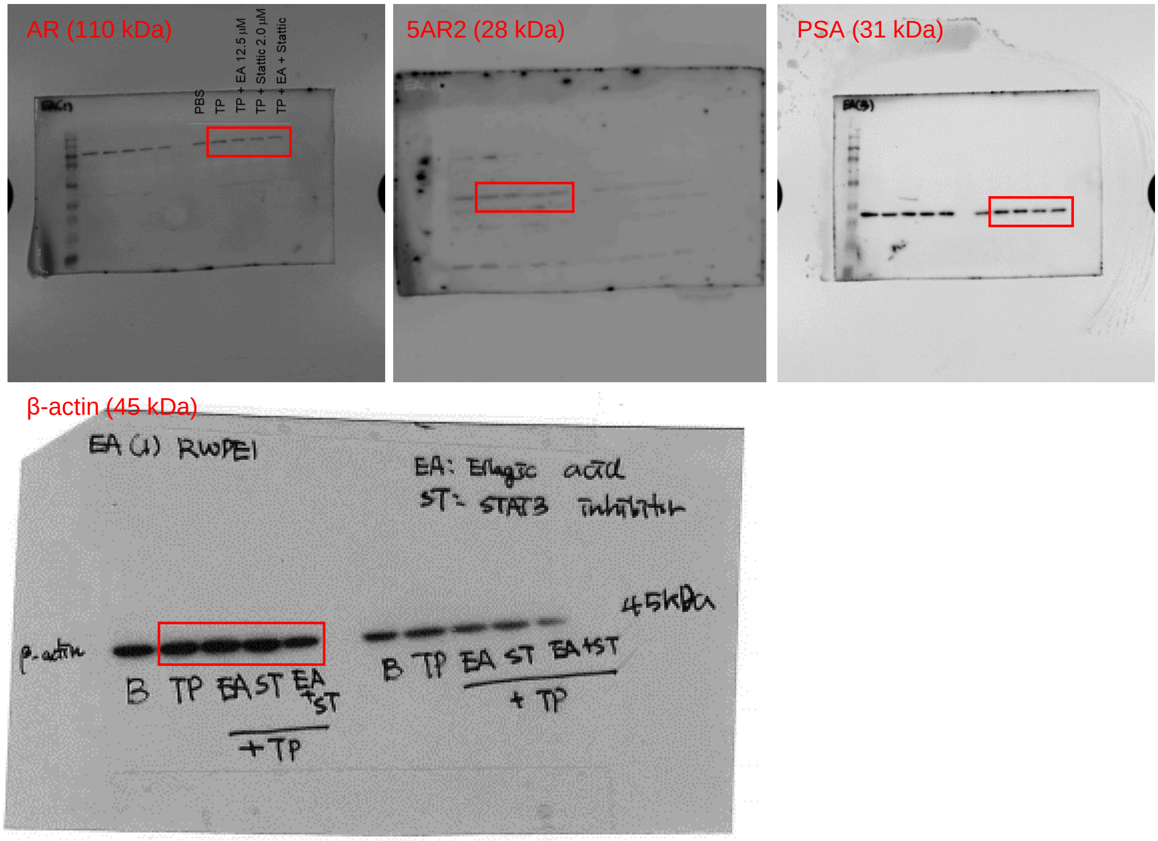

Figure 6f

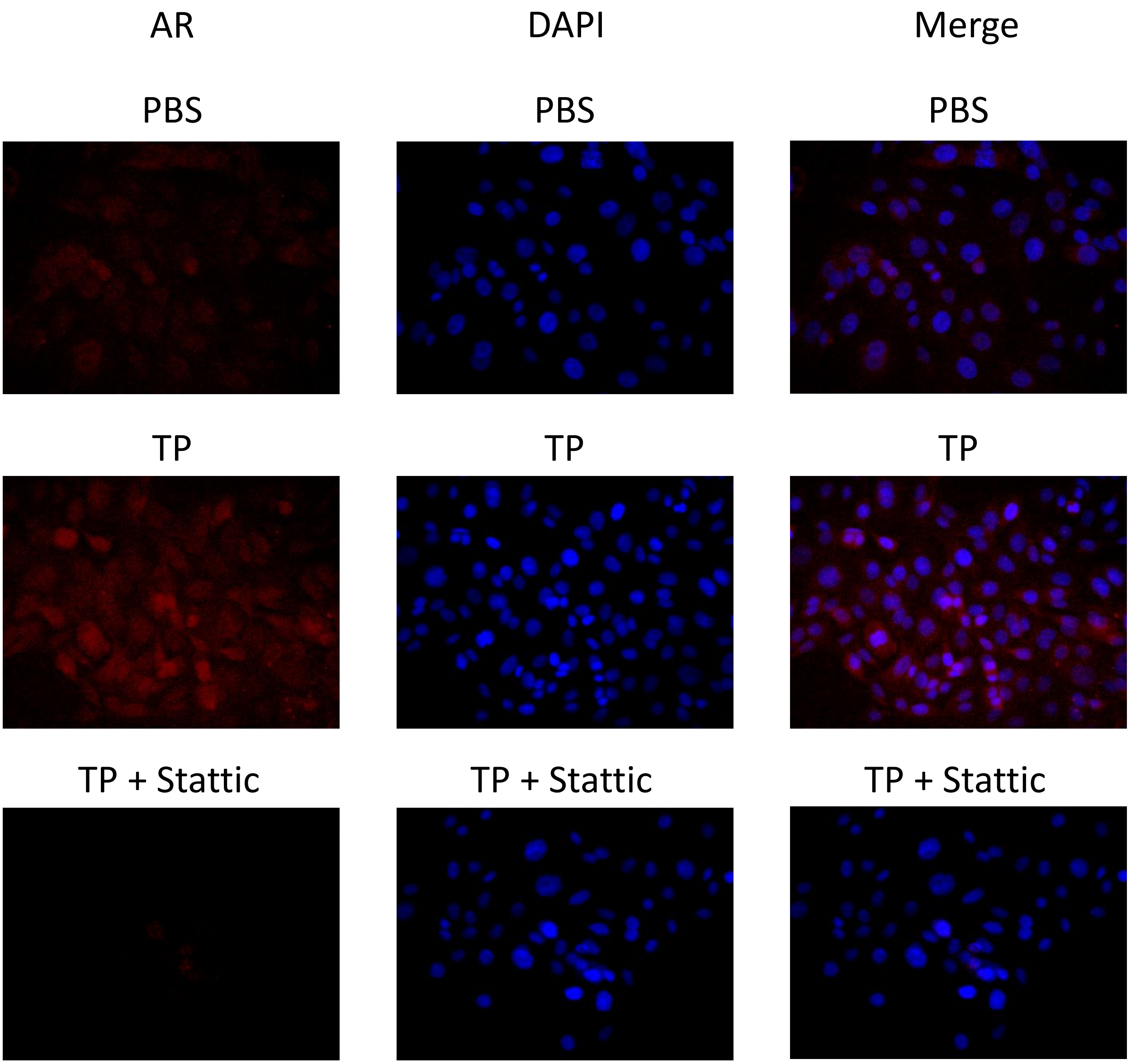

X400
